# Uncovering the Protective Mechanism of the Volatile Oil of *Acorus tatarinowii* against Acute Myocardial Ischemia Injury Using Network Pharmacology and Experimental Validation

**DOI:** 10.1155/2021/6630795

**Published:** 2021-06-22

**Authors:** Zhen-Zhong Zang, Li-Mei Chen, Yuan Liu, Yong-Mei Guan, Qing Du, Pan Xu, Qian Shen, Ming Yang, Hong-Ning Liu, Zheng-Gen Liao

**Affiliations:** ^1^Key Laboratory of Modern Preparation of Chinese Medicine, Ministry of Education, Jiangxi University of Chinese Medicine, Nanchang, Jiangxi, China; ^2^Jiangxi Province Key Laboratory of Chinese Medicine Etiopathogenisis, Jiangxi University of Chinese Medicine, Nanchang, Jiangxi, China; ^3^The Affiliated Hospital of Jiangxi University of TCM, Nanchang, Jiangxi, China

## Abstract

*Acorus tatarinowii* is a traditional aromatic resuscitation drug that can be clinically used to prevent cardiovascular diseases. The volatile oil of *Acorus tatarinowii* (VOA) possesses important medicinal properties, including protection against acute myocardial ischemia (MI) injury. However, the pharmacodynamic material basis and molecular mechanisms underlying this protective effect remain unclear. Using network pharmacology and animal experiments, we studied the mechanisms and pathways implicated in the activity of VOA against acute MI injury. First, VOA was extracted from three batches of *Acorus tatarinowii* using steam distillation, and then, its chemical composition was determined by GC-MS. Next, the components-targets and protein-protein interaction networks were constructed using systematic network pharmacology. Gene Ontology (GO) function and Kyoto Encyclopedia of Genes and Genomes (KEGG) pathway enrichment analyses were also conducted in order to predict the possible pharmacodynamic mechanisms. Furthermore, animal experiments including ELISAs, histological examinations, and Western blots were performed in order to validate the pharmacological effects of VOA. In total, 33 chemical components were identified in VOA, and *ß*-asarone was found to be the most abundant component. Based on network pharmacology analysis, the therapeutic effects of VOA against myocardial ischemia might be mediated by signaling pathways involving COX-2, PPAR-*α*, VEGF, and cAMP. Overall, the obtained results indicate that VOA alleviates the pathological manifestations of isoproterenol-hydrochloride-induced myocardial ischemia in rats, including the decreased SOD (superoxide dismutase) content and increased LDH (lactic dehydrogenase) content. Moreover, the anti-MI effect of VOA might be attributed to the downregulation of the COX-2 protein that inhibits apoptosis, the upregulation of the PPAR-*α* protein that regulates energy metabolism, and the activation of VEGF and cAMP signaling pathways.

## 1. Introduction

Cardiovascular diseases are the leading cause of morbidity and mortality in the world. In China, the prevalence of cardiovascular diseases is increasing year after year. Myocardial ischemia (MI), one of the main pathogenic mechanisms of cardiovascular diseases [1, 2], is a pathological state characterized by reduced blood perfusion and oxygen supply to the heart. As a metabolic disease, this state leads to abnormal myocardial energy metabolism and disrupts normal heart function. Patients suffering from mild MI have angina pectoris and arrhythmia; whereas, those suffering from the severe disease exhibit myocardial infarction, which eventually leads to death [3]. Acute ischemia, hypoxia, and metabolic disorders in the myocardial tissues are caused by severe spasms, sudden coronary artery obstruction, low blood pressure, reduced aortic blood supply, change in blood viscosity, valvular disease, and myocardial disease [4, 5].

The genus Acorus belongs to the Acoraceae family that is widely distributed in temperate and subtropical regions, and it includes *Acorus tatarinowii* (shi chang pu), *Acorus calamus* L (shui chang pu), *Acorus gramineus* Aiton (jin xian pu), and *Acorus macrospadiceus* F. N. Wei (shannai chang pu) species [6, 7]. Although *Acorus tatarinowii* is similar to *Acorus calamus* L, the former is smaller in terms of morphology, and its leaves lack a midrib. Moreover, *Acorus tatarinowii* is more frequently used for medicinal purposes [7], and it is recognized as a traditional aromatic resuscitation medicine that eliminates phlegm, refreshes the mind, nourishes intelligence, dispels dampness, and stimulates appetite [8, 9]. The volatile oil of *Acorus tatarinowii* (VOA) is one of the ingredients responsible for its medicinal properties. According to previous pharmacological studies, VOA can be used to treat or prevent cardiovascular diseases [10–14]. The chemical composition of VOA is complex, and so far, more than 60 components have been identified [15], including phenylpropanoids (*α*-asarone, *ß*-asarone, methyl eugenol, isomethyl eugenol, and elemene), monoterpenoids (borneol, camphor, *α*-pinene, *ß*-pinene, and kenene), and sesquiterpenoids (*β*-elemene, *ß*-syringene, and colicone) [16]. Among these various components, *aα*-asarone and *ß*-asarone are primarily responsible for the cardiovascular system-protection activity of VOA [17–21]. In particular, *α*-asarone and *ß*-asarone reduce the endothelin (ET) level and increase the NO content. Moreover, they decrease plasma viscosity and necrosis in rats with myocardial ischemia, improve the blood flow in rats with hyperviscosity, and reduce blood lipid levels in atherosclerotic rats, which proves that they can protect the cardiovascular system [14,22].

Although VOA protects against acute MI injury, its pharmacodynamic material basis and the underlying molecular mechanisms have not yet been elucidated. In this study, we explore these mechanisms using network pharmacology and animal experiments. Network pharmacology is a tool used to systematically study multicomponent drug mechanisms by analyzing the relationships between drugs, compounds, drug targets, diseases, and pathways [23, 24]. Considering that traditional Chinese medicine (TCM) involves many small compounds that may simultaneously, transiently, or weakly bind with multiple target proteins [25, 26], it is important to analyze the effects of herbal treatments on various diseases using a holistic approach. The holistic perspective of TCM is consistent with the key idea of network pharmacology [27], wherein the potential mechanisms of active TCM components are systematically elaborated by constructing molecular networks [28, 29].

## 2. Materials and Methods

### 2.1. Materials

*Acorus tatarinowii*, propranolol hydrochloride tablets, and isoproterenol hydrochloride injection were purchased from Hebei Jinye Pharmaceutical Co. Ltd. (Hebei, China), Jiangsu Yabang Epson Pharmaceutical Co. Ltd. (Jiangsu, China), and Shanghai Wellhope Pharmaceutical Co. Ltd. (Shanghai, China), respectively. SOD, LDH, COX-2, and PPAR*α* ELISA kits were obtained from Jiangsu Meimian Industry Co., Ltd. (Jiangsu, China), while COX-2 antibodies and GADPH antibodies were purchased from Abcam (Cambridge, England). PPAR-*α* antibodies were bought from Beijing Boaosen Biotechnology Co. Ltd. (Beijing, China).

### 2.2. Extraction of the Volatile Oil

*Acorus tatarinowii* was verified by Associate Professor Kezhong Deng and deposited in the key laboratory of modern preparation at Jiangxi University of Traditional Chinese Medicine. Three hundred grams of the rhizome of *Acorus tatarinowii* (190127c103, Hebei, China) were weighed and placed in a round bottom flask, followed by soaking in water for 30 min. The volatile oil in the rhizome was extracted by steam distillation for 9 h, and then, it was dehydrated with anhydrous sodium sulfate. After measuring the volume of the dehydrated oil, the sample was transferred to a brown bottle and stored at 4°C.

### 2.3. Quality Control

Fingerprint technology is often used to control the quality of TCM plants. Herein, we extracted the volatile oils of three batches of *Acorus tatarinowii* (S1: 170801c103, S2: 181001c103, and S3: 190127c103) and established the fingerprint of VOA for quality control.

### 2.4. Composition of VOA

To determine the chemical composition of VOA, 10 mg of the VOA was added to methanol (181128, Guangzhou, China) in a 25 mL volumetric flask. After adjusting the volume and homogenizing the mixture, 25 *μ*L of the resulting solution was diluted with methanol in a 10 mL volumetric flask. The obtained test solution was analyzed by gas chromatography/mass spectrometry using a DB-624 elastic quartz capillary column (30 m × 320 *μ*m × 1.8 *μ*m) (Agilent, USA) and an EI ion source. Chromatographic separation was achieved using a 1 mL/min flow of helium gas, and the injection port temperature and pressure were set at 260°C and 55.3 kPa, respectively. The split ratio of the injected sample (1 *μ*L) was maintained at 20 : 1. The temperature of the column was programmed as follows: conditioning at 50°C for 3 min, run at the initial temperature of 70°C, and increase temperature to 120°C at the rate of 10°C/min (held for 0 min) and then to 265°C at the rate of 5°C/min (held for 3 min). The temperature of the ion source was set at 230°C, whereas that of the quadrupole was 150°C. The electron energy, multiplier voltage, solvent delay time, and mass range were 70 eV, 2.46 kV, 3 min, and 30–650, respectively.

### 2.5. Screening of Targets Related to the Chemical Constituents and MI

The chemical structures and SMILES strings of the identified VOA components were taken from PubChem (https://pubchem.ncbi.nlm.nih.gov/) and TCMSP (http://lsp.nwu.edu.cn/tcmsp.php/). The SMILES strings of the compounds were entered into the SwissTargetPrediction (http://www.swisstargetprediction.ch/) and STITCH (http://stitch.embl.de/) servers to determine the potential targets. After listing all targets, duplicates were removed, and the final potential targets of the VOA were obtained. The targets related to myocardial ischemia were obtained from the OMIM (https://www.omim.org/) and DisGeNET (http://www.disgenet.org/) databases using the phrase “myocardial ischemia” as search keyword. After listing all targets, duplicates were removed, and the final list of targets was obtained.

### 2.6. Construction of the Components-Targets Network and Protein-Protein Interaction (PPI) Network

First, the Venny 2.1.0 online software (https://bioinfogp.cnb.csic.es/tools/venny/index.html) was used to identify the overlapping targets amongst the lists of targets related to VOA components and myocardial ischemia disease. Then, a complex network was built between the components and overlapping targets. Protein-protein interaction (PPI) data were obtained from the STRING 11.0 database (https://string-db.org/). The overlapping targets were searched using the “multiple proteins” option and “*Homo sapiens*” organism, and the data were imported into Cytoscape 3.7.1 to build the PPI network.

### 2.7. Construction of the Organs-Targets Network

The BioGPS (http://biogps.org/#goto=welcome) database was used to determine the distribution of different targets in tissues or organs. The top ten tissues or organs were screened with gene expression, and the screening results were imported into Cytoscape 3.7.1 to build the overlapping targets-tissues/organs distribution network.

### 2.8. GO Function and KEGG Pathway Enrichment

The ClusterProfiler software package in the R-Studio platform was used to perform the GO function and KEGG pathway enrichment analyses of the coacting targets. In order to find the GO functions and KEGG pathways that are significantly enriched in the overlapping targets, the significance level was set to *p* *<* 0.05.

### 2.9. Molecular Docking

Through molecular docking of chemical components and targets, the reliability of prediction results of potential targets can be further verified. The Discovery Studio 4.5 Client software was used to conduct molecular docking of chemical components of VOA and targets, and the docking score of the molecular docking results was analyzed to evaluate the degree of binding between the chemical components of VOA. The positive drug propranolol hydrochloride was also analyzed for molecular docking with targets.

### 2.10. Experimental Validation

#### 2.10.1. Animals

Specific pathogen free (SPF) grade male Kunming mice, weighing 20 ± 2 g, were provided by the Experimental Animal Science and Technology Center of Jiangxi University of Traditional Chinese Medicine (license number: SCXK (Gan) 2018–0003). All mice were kept at temperatures of 18–25°C and provided with water. The animal procedures performed herein were all approved by the Ethics Committee of the Experimental Animal Science and Technology Center (NO. JZLLSC2019-0139).

#### 2.10.2. Induction of Acute MI Injury

The mice were randomly divided into six groups, with eight mice in each group: (1) control group, (2) acute MI injury model group (model group), (3) low dose of the VOA group (55 mg kg^−1^ d^−1^), (4) medium dose of the VOA group (110 mg kg^−1^ d^−1^), (5) high dose of the VOA group (220 mg kg^−1^ d^−1^), and (6) propranolol group (E180913, Jiangsu, China) (48 mg kg^−1^ d^−1^). The mice in groups (3)–(6) were administered with VOA by gavage once a day for 17 consecutive days. Meanwhile, the mice in groups (1) and (2) were given equivalent volumes of normal saline (190104, Guangzhou, China). The animal model of acute MI injury was established using previously reported methods [30, 31]. In brief, all mice, except for control group mice, were intraperitoneally injected with isoproterenol hydrochloride (190104, Shanghai, China) (10 mg kg^−1^ d^−1^) on day 15 for three consecutive days. The injections were administered one hour after intragastric VOA ingestion. Instead of isoproterenol hydrochloride, the mice in the control group were intraperitoneally injected with the same volume of normal saline.

#### 2.10.3. Sample Collection

On the last day of the experiment, the mice were sacrificed under pentobarbital sodium anesthesia, and blood samples were collected and centrifuged at 3500 r/min for 15 min. The supernatants were stored at −80°C for further analysis. The heart was quickly dissected, and some heart tissues were put in 10% paraformaldehyde solution for histopathological examination. The remaining tissues were stored in a refrigerator at −80°C.

#### 2.10.4. Determination of SOD and LDH Concentrations in Serum and Histological Examination

The biochemical indexes of serum SOD and LDH (1911M, MEIMIAN, Jiangsu, China) were determined as the instructions. The myocardial tissues were fixed with 10% formaldehyde solution for 48 h, embedded in paraffin, cut into 4 mm sections, and stained with hematoxylin and eosin. The histological images were studied under light microscopy (200×).

#### 2.10.5. ELISA Measurements of COX-2 and PPAR-*α* Levels

The COX-2 and PPAR-*α* levels in the myocardium were measured using the COX-2 mouse ELISA kit (MM-0356M1, Jiangsu, China) and PPAR-*α* mouse ELISA kit (MM-0249M1, Jiangsu, China), respectively.

#### 2.10.6. Western Blot Analysis

To determine the COX-2 and PPAR-*α* protein contents in the myocardial tissues of mice, a specific amount of the tissues was placed in a tissue homogenizer for full homogenization. Afterward, RIPA (Solaibao, Beijing, China) was added to completely lyse the tissues. Subsequently, the lysed samples were centrifuged at 12000 g and 4°C for 15 min, and then, the supernatants were collected for protein quantification using the BCA protein analysis kit (PICPI23223, Thermo, Waltham, USA). The proteins were separated on a 10% resolution SDS-PAGE gel (S1010, Solaibao, Beijing, China) and then transferred to a nitrocellulose membrane (HATF00010, Millipore, Waltham, USA). In turn, the membrane was blocked with 5% skimmed milk and incubated overnight with antibodies on a 4°C cradle. The antibodies used for Western blotting include anti-COX-2 (1 : 1000, ab15191, Abcam, Cambridge, England), anti-PPAR-*α* (1 : 800, Bs23398 R, Bioss, Beijing, China), and anti-GAPDH (1 : 2500, ab9485, Abcam, Cambridge, England) antibodies. Afterward, the membrane was incubated with HRP-labeled secondary antibodies (1 : 1000, A0208, Beyotime, China) at 37°C for 1 h. As per the required dosage, the ECL (WBKLS0100, Millipore, USA) luminescent solutions A and B were mixed evenly and added to the front of the membrane. The membrane was kept in the dark for 5 min, and then, the developer was poured out and carefully absorbed with paper. Finally, the membrane was covered with a layer of flat transparent paper and scanned using the imaging system (Tanon-5200, Tanon, China).

### 2.11. Statistical Methods

The data were statistically analyzed using the SPSS 17.0 software (Armonk International Business machines, New York, USA), and they are expressed as mean ± standard deviation (SD). Student's *t*-test was used to compare different groups, and *p*-values < 0.05 were considered to be statistically significant.

## 3. Results

### 3.1. Fingerprint Analysis of VOA

**Figure 1:**
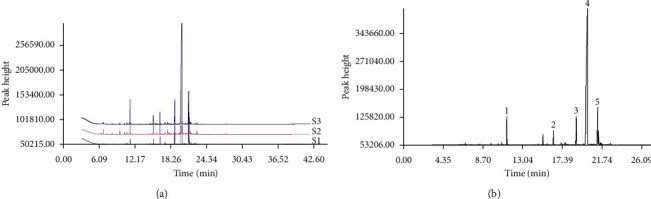
(a) Three batches of VOA fingerprints. (b) Total ion flow chromatogram of VOA (1, estragole; 2, methyl isoeugenol; 3, benzene, 1,2,3-trimethoxy-5-(2-propenyl)-; 4, *ß*-asarone; 5, *α*-asarone).

The GC-MS data of three VOA batches were imported into the Chinese medicine chromatographic fingerprint similarity evaluation system version A (2004) for similarity evaluation, and the S1 chromatogram was used as a reference spectrum. After multipoint calibration and automatic matching, the control fingerprint was acquired (Figure 1(a)). Based on the obtained results, the fingerprints of all three VOA batches are similar, and they have a common pattern.

### 3.2. Identification of VOA Constituents

The total ion flow chromatogram of VOA is shown in Figure 1(b). In total, 33 compounds were identified in VOA using GC-MS analysis (Table 1), with *ß*-asarone, *α*-asarone, benzene, 1,2,3-trimethoxy-5-(2-propenyl), methyl isoeugenol, and estragole accounting for 93.7% of the total composition.

### 3.3. Targets Related to the Chemical Constituents and to MI

All targets retrieved from SwissTargetPrediction and STITCH databases were integrated, and the duplicates were removed. Overall, 589 (Supplementary Table S1) and 600 (Supplementary Table S2) targets related to the 33 VOA components and to the MI disease were obtained. Note that the disease targets were obtained through OMIM and DIGENET databases.

### 3.4. The Components-Targets Network

**Figure 2:**
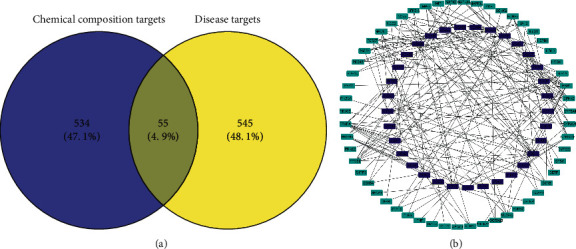
(a) Venny diagram of component targets and disease targets and (b) the “components-targets” network. The 33 blue nodes represent the components of VOA, the 55 green nodes represent the overlapping targets, and the lines represent relationships. The edges denote that nodes can interact with each other.

Figure 2(a) shows the 589 target symbols of the drug and the 600 gene symbols of the disease, 55 overlaps. These overlapping targets may be critical for VOA-mediated MI treatment. Supplementary Table S3 provides details regarding the 55 overlapping targets.

To assess how VOA may act against myocardial ischemia, the Cytoscape 3.7.1 software was used to build a “components-targets” network, as shown in Figure 2(b). The 33 blue nodes represent the VOA components, and the 55 green nodes represent the overlapping targets. The components-targets network analysis showed that the targets were highly correlated with multiple chemical components. For instance, ESR1 was associated with 16 components, PPAR-*α* with 12, and P2RX7 with 10. The key enzyme in prostaglandin biosynthesis, PTGS (also known as cyclooxygenase (COX)), was also correlated with 9 components. Therefore, inducible COX-2 was associated with nine components. Moreover, 24 chemical components were related to multiple targets. For example, *α*-asarone, *ß*-asarone, and artemisinol were associated with 19, 18, and 12 targets, respectively, and elemene, neroli, and methyl isoeugenol were all associated with 13 targets. It should be noted that the targets showing strong correlation with chemical components may play a significant role in the anti-MI activity of VOA. Detailed information regarding the chemical components and targets is given in Supplementary Tables S4 and S5.

### 3.5. PPI Network

**Figure 3:**
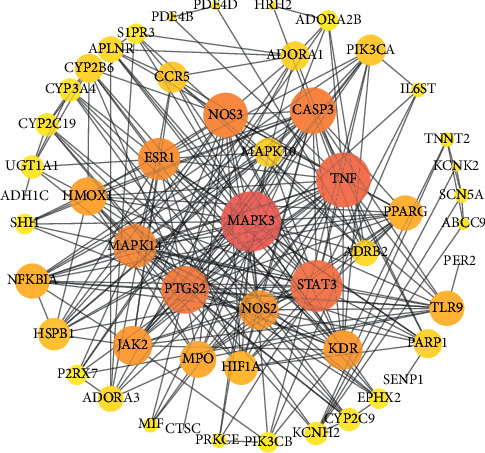
Interaction network between overlapping targets. The node size and color depth represent the value of degree. The lines represent the relationship between overlapping targets.

As shown in Figure 3, the network graph comprises 55 nodes and 266 edges. In general, large degrees of freedom indicate high action intensity of the potential target. When the node color changes from dark pink to yellow, the degree value becomes small. The edges represent correlations between the overlapping targets. The greater the binding degree between the targets, the larger the binding score, and the more coarse the edge. Considering that the connecting edges of MAPK3 with STAT3, TNF with NFKBIA, STAT3 with JAK2, Ll6ST, and HIF1A, PTGS2 with STAT3, and CASP3 with PARP1 and MAPK14 are thicker than other edges, these targets showed greater degree of binding. Moreover, the MAPK3, TNF, STAT3, PTGS2, and CASP3 targets may play an important role in the anti-MI activity of VOA. Based on network analysis, the average local clustering coefficient was 0.623, and the average node degree was 9.67.

### 3.6. Construction of the Organs-Targets Network

**Figure 4:**
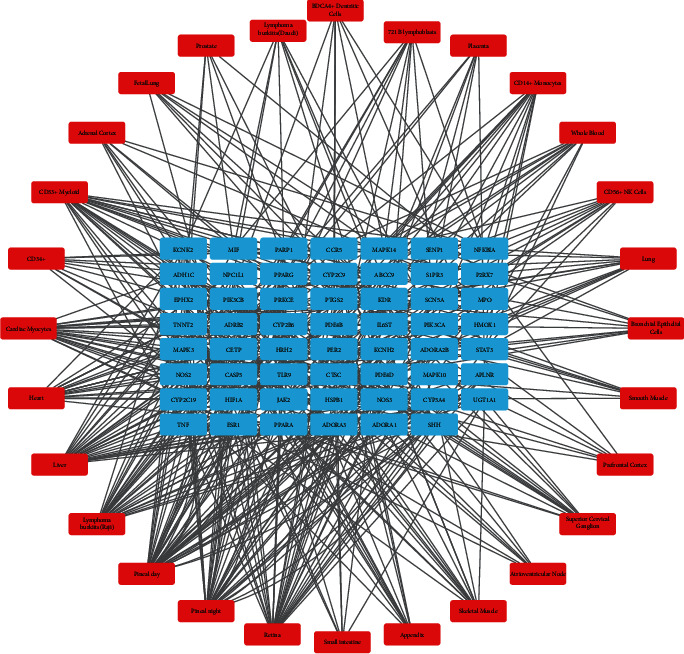
Overlapping targets-tissues/organs network. The blue nodes represent the targets, the red nodes represent the tissues/organs, and the lines represent the relationships between targets and tissues/organs.

In Figure 4, blue color represents the potential targets of VOA, whereas red color represents the tissues/organs in which these targets were distributed. The edges correspond to relationships between the targets and tissues/organs. Overall, 22 targets were distributed in the heart, and 22 genes were highly expressed in the cardiac myocytes. Moreover, many targets were distributed in the blood, liver, and retina. The genes of antigens CD33, CD14, CD56, and CD34 were also present.

### 3.7. GO and KEGG Analyses

**Figure 5:**
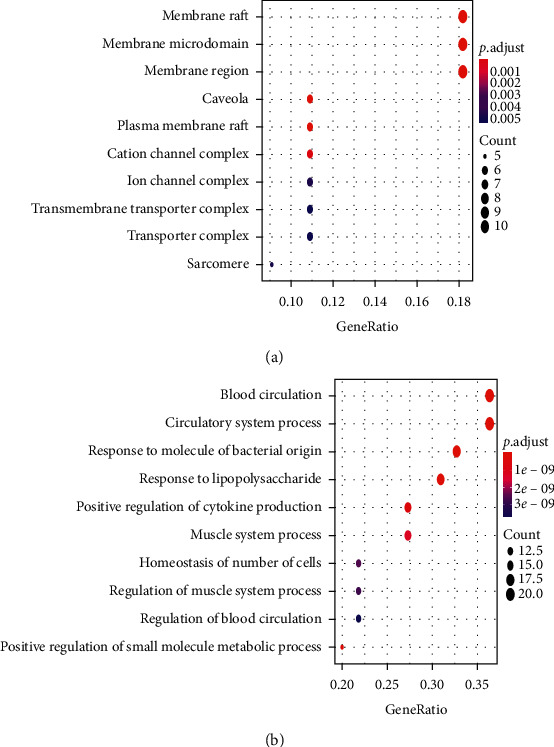
(a) The BP enriched network of overlapping targets. (b) The CC enriched network of overlapping targets.

**Figure 6:**
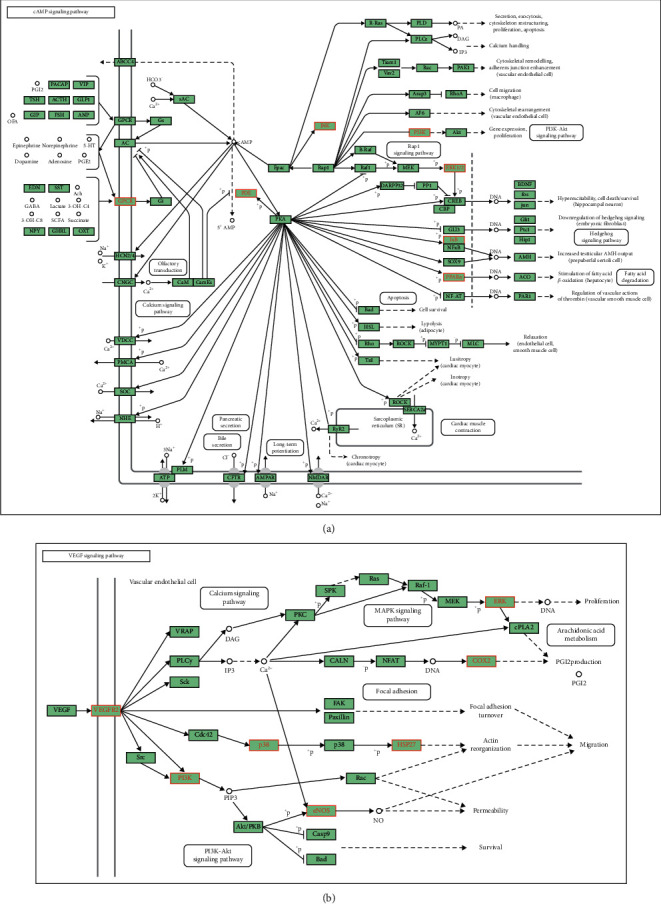
(a) The diagram of the VEGF signaling pathway (red font as key targets). (b) The diagram of the cAMP signaling pathway (red font as key targets). The pathway map was downloaded from KEGG website.

GO and KEGG analyses were used to assess the biological characteristics of the potential VOA targets. The results reveal that the top predictors in biological processes (BP) include blood circulation, circulatory system processes, responses to molecules of bacterial origin and lipopolysaccharides, positive regulation of small-molecule metabolism and cytokine production, and blood circulation regulation. Therefore, VOA might play a role in the treatment of myocardial ischemia by improving these biological processes (Figure 5(a)). The biological processes related to PTGS2 and PPAR-*α* targets were blood circulation, circulatory system processes, and positive regulation of small-molecule metabolism. Hypothetically, these processes might play an important role in the treatment of myocardial ischemia by VOA. In terms of cellular components (CC), the top predictors were membrane raft, membrane microdomain, membrane region, membrane invagination, membrane raft plasma, ion channel complex, and transmembrane transporter complex (Figure 5(b)). The targets of PTGS2, TNF, NOS3, JAK2, HMOX1, and MAPK3 were related to membrane raft, membrane microdomain, and membrane region. Based on KEGG pathway enrichment analysis, the overlapping targets identified herein are implicated in 118 pathways that are mainly related to the metabolism, inflammation, immune function, and endocrine system. The top 20 KEGG pathways include AGE-RAGE (pathway involved in diabetic complications), sphingolipid, VEGF, prolactin, and cAMP signaling pathways. Supplementary Table S6 provides further details regarding these pathways.  Figure 6 shows the pathway diagrams of VEGF and cAMP signaling generated in *R* Studio.

### 3.8. Molecular Docking

The top 5 targets with degree values in the “components-targets” network were selected for molecular docking verification. The 33 chemical components of the VOA and ESR1, PPAR-*α*, P2RX7, PTGS2, and EPHX2 were import into Discovery Studio 4.5 client for molecular docking. It is generally believed that docking score value greater than 100.0 indicates a strong binding activity between the molecule and the target, a value greater than 80.0 indicates a good binding activity, and a value above 60.0 indicates a certain binding activity between the molecule and the target.  Table 2 provides that 28 chemical components in the VOA had good binding activity with important targets, and the docking of the positive drug propranolol hydrochloride with the target had a high score, which further explained the importance of ESR1, PPAR-*α*, P2RX7, PTGS2, and EPHX2.

### 3.9. Experimental Validation

#### 3.9.1. Serum Levels of SOD and LDH

**Figure 7:**
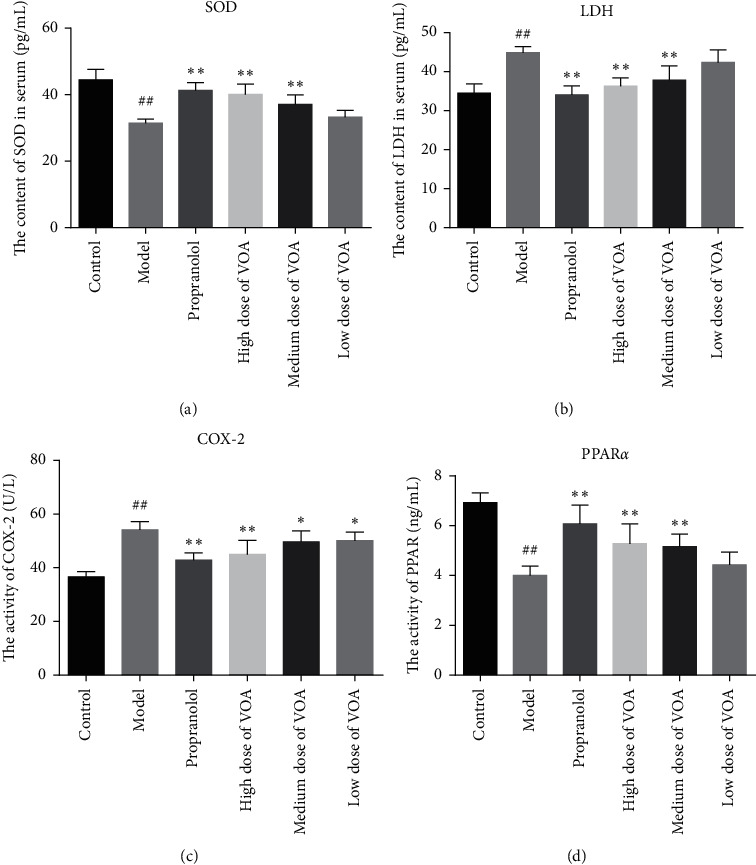
(a) The contents of SOD in serum (‾*x* ± *s*, *n* = 8). (b) The contents of LDH in serum (‾*x* ± *s*, *n* = 8). (c) The activity of COX-2 in myocardial tissue (‾*x* ± *s*, *n* = 8). (d) The activity of PPAR-*α* in myocardial tissue (‾*x* ± *s*, *n* = 8). Compared with the control group: ^*#*^*p* *<* 0.05 and ^##^*p* *<* 0.01; compared with the model group: ^*∗*^*p* *<* 0.05 and ^*∗∗*^*p* *<* 0.01.

Compared to the control group mice, the SOD level in the model group mice is significantly reduced (*p* *<* 0.01), while the LDH level was significantly increased (*p* *<* 0.01). This indicated that intraperitoneal injection of isoproterenol hydrochloride (10 mg kg^−1^ d^−1^) damages the hearts of mice, resulting in a successful MI model. Moreover, the medium dose of VOA, high dose of VOA, and propranolol groups exhibit significantly increased SOD levels (*p* *<* 0.01) and significantly reduced LDH levels (*p* *<* 0.01) compared to the control group. However, the SOD and LDH levels in low dose of VOA were comparable to those determined for the model group (*p* *>* 0.05) (Figures 7(a) and 7(b)).

#### 3.9.2. ELISA

The concentrations of COX-2 in the myocardium of the model group mice were significantly greater than that in the control group mice (*p* < 0.01). The concentrations of PPAR-*α* in the myocardium of the model group mice were significantly lower than that in the control group mice (*p* *<* 0.01). Compared to the model group, the concentration of COX-2 in the medium dose of VOA, high dose of VOA, and propranolol groups were appreciably reduced (*p* *<* 0.01), whereas that of PPAR-*α* was increased (*p* < 0.05). Similarly, treatment with low dose of VOA reduced the concentration of COX-2 (*p* < 0.05) in the myocardium and increased the concentration of PPAR-*α* (*p* > 0.05). Overall, these results indicated that VOA alleviates MI injury in mice (Figures 7(c) and 7(d)).

#### 3.9.3. Histological Examination

**Figure 8:**
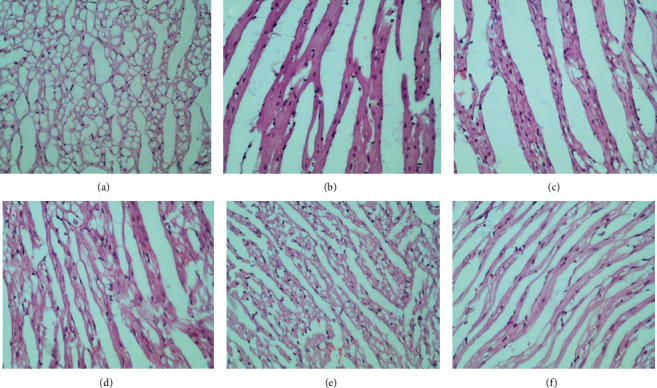
The H&E staining results in different groups × 200. (a) Control group, (b) model group, (c) low dose of the VOA group, (d) medium dose of the VOA group, (e) high dose of the VOA group, and (f) propranolol group.

The histological examination of myocardial fibers collected from the model group mice showed that these fibers were arranged in a disordered manner and that the myocardial tissues exhibit breaks, inflammatory cell infiltration, degeneration, and necrosis. However, VOA and propranolol hydrochloride treatments can improve the degree of myocardial injury in mice suffering from isoproterenol-hydrochloride-induced acute myocardial ischemia (Figure 8).

#### 3.9.4. Western Blot Analysis

**Figure 9:**
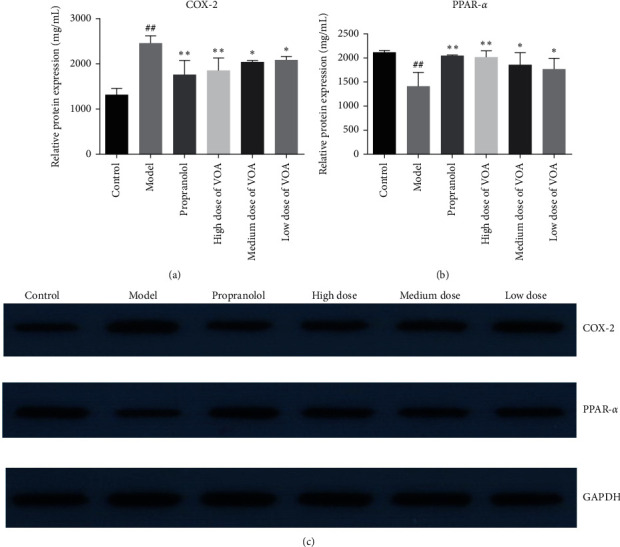
Western blot analysis of COX-2 and PPAR-*α* in myocardial tissue of rats in each group (‾*x* ± *s*, *n* = 3) (compared with the control group: ^#^*p* < 0.05 and ^##^*p* < 0.01; compared with the model group: ^*∗*^*p* < 0.05 and ^*∗∗*^*p* < 0.01).

Compared to the control group, the expression of the COX-2 protein in the model group was significantly increased (*p* < 0.01), whereas that of the PPAR-*α* protein is significantly reduced (*p* < 0.01). However, treatment with low, medium, or high dose of VOA decreased the expression of COX-2 (*p* < 0.05, *p* < 0.01) and increased that of PPAR-*α* (*p* < 0.05, *p* < 0.01). Overall, the results indicated that VOA can improve acute MI injury in mice and that its effect was dose-dependent (Figure 9).

## 4. Discussion

In addition to the phenylpropanoid main active components, the volatile oil of *Acorus tatarinowii* (VOA) contains monoterpenes, sesquiterpenes, aliphatic aldehydes, and ketones [32]. In this study, we show that of all the components in VOA, *ß*-asarone, *α*-asarone, elemene, nerol, and methyl isoeugenol have the most targets. Except for neroli, which is a sesquiterpenoid, these compounds belong to the phenylpropanoid family. In general, phenylpropanoid compounds are known to protect the cardiovascular system by causing vasorelaxation and antiplatelet aggregation and by lowering the blood lipid level and the blood pressure. Moreover, they protect against ischemia/reperfusion injury and myocardial hypertrophy [33]. *ß*-Asarone has a variety of pharmacological effects, including myocardial cell protection [34, 35], anti-inflammation [36], vascular endothelial cell protection [37], antiplatelet aggregation and adhesion [38], and antiischemia [39]. Similarly, *α*-asarone exhibits antithrombosis, antiplatelet aggregation, and antihyperlipidemia activities, among others. As for neroli, it has antioxidation, anti-inflammatory, antibiofilm, skin penetration, antiulcer, antiworm, and anticancer effects [40]. By resisting the influence of reactive oxygen species [41–43], neroli protects the cells from the oxidative damage induced by lipids, proteins, and DNA. The “components-targets” network constructed in this study reveals that the degrees of ESR1, PPAR-*α*, P2RX7, and PTGS2 are greater than those of other targets. Therefore, these targets may play a critical role in the anti-MI effect of VOA. As a ligand-activated transcription factor, ESR1 mainly mediates the biological effects of estrogen and its receptor modulators [44], and it also affects cell proliferation and differentiation in target tissues. ESR1 plays a vital role in maintaining the homeostasis of cardiomyocytes, regulating vasodilation, reducing cardiomyocyte apoptosis, and stimulating the formation of new blood vessels [45]. Meanwhile, PPAR-*α* is involved in the regulation of several bodily functions, including lipid metabolism, cell proliferation, and adhesion, and it is also implicated in pathways related to cytokines and inflammatory factors. The ligands of PPAR-*α* cure cardiovascular diseases and alleviate their complications. They also reduce the area of myocardial necrosis, improve ischemia and the function of posterior myocardial contraction, protect against acute myocardial injury, and enhance myocardial antiischemic ability [46]. As a member of the purinergic receptor family, P2RX7 is activated by extracellular ligands (such as ATP), and it participates in cell signal transduction, cytokine secretion, and mediation of cell growth, among other biological functions [47]. The activation of P2RX7 can cause microvascular dysfunction and local hypoxia [48]. As for PTGS2 (also known as COX-2), it is responsible for producing inflammatory prostaglandins [49, 50]. According to previous reports, COX-2 inhibitors have a protective effect against myocardial ischemic injury in adult rabbits [51].

Based on KEGG enrichment analysis, the AGE-RAGE, sphingolipid, VEGF, and cAMP signaling pathways are closely related to the anti-MI properties of VOA. AGEs can reduce blood vessel elasticity and NO content, resulting in damaged vascular endothelial cell function [52]. The combination of AGEs and receptor RAGE promotes the expression of a series of atherosclerosis-related genes such as vascular cell adhesion molecule-1, tissue factor, and monocyte chemoattractant protein-1 [53]. The sphingomyelin signaling pathway may also be implicated in the pathophysiological mechanism of ischemia-reperfusion injury [54]. Ginsenoside F11 has anti-MI properties, and the underlying mechanism of action may involve the regulation of multiple signaling pathways such as sphingomyelin metabolism, arachidonic acid metabolism, and linoleic acid metabolism [55]. MicroRNA-320a mediates doxorubicin myocardial injury through targeted inhibition of the VEGF signaling pathway. The inhibition of microRNA-320a expression can alleviate cardiac injury [56].

## 5. Conclusions

In conclusion, this study shows that VOA can protect against myocardial ischemia by regulating biological processes such as blood circulation, small-molecule metabolism, and cytokine production. The protective effect of VOA is associated with several targets, mainly COX-2, PPAR-*α*, and ESR1, that are involved in the AGE-RAGE, sphingolipid, VEGF, and cAMP signaling pathways. Considering that COX-2 is a key target in the VEGF signaling pathway and that PPAR-*α* is a key target in the cAMP signaling pathway, the anti-MI mechanism of VOA may involve both pathways. Moreover, *ß*-asarone and *α*-asarone may be the main components in VOA responsible for its protective effect against myocardial ischemia.

## Figures and Tables

**Table 1:** Chemical constituents and relative percentage content of VOA.

Number	Chemical composition	Structure	Molecular formula	Relative percentage content
VOA1	*α*-Pinene	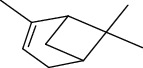	C_10_H_18_	0.12
VOA2	Camphene	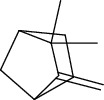	C_10_H_16_	0.29
VOA3	*β*-Pinene	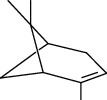	C_10_H_18_	0.11
VOA4	*o*-Cymene	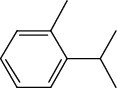	C_10_H_14_	0.06
VOA5	*D*-Limonene	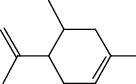	C_10_H_16_	0.07
VOA6	*γ*-Terpinene	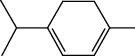	C_10_H_16_	0.05
VOA7	Camphor	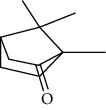	C_10_H_16_O	0.25
VOA8	endo-Borneol	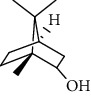	C_10_H_18_O	0.32
VOA9	Estragole	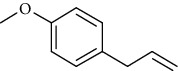	C_10_H_12_O	3.50
VOA10	Longicyclene	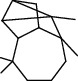	C_15_H_24_	0.19
VOA11	Caryophyllene	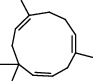	C_15_H_24_	0.24
VOA12	*γ*-Muurolene	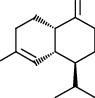	C_15_H_24_	0.40
VOA13	1H-Cyclopropa[a]naphthalene, 1a,2,3,5,6,7,7a,7b-octahydro-1,1,7,7a-tetramethyl-, [1aR-(1a.alpha.,7.alpha.,7a.alpha.,7b.alpha.)]-	—	C_15_H_24_	0.11
VOA14	Methyl isoeugenol	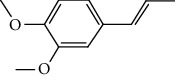	C_11_H_14_O_2_	2.95
VOA15	1,3,5-Cycloheptatriene, 2,4-diethyl-7,7-dimethyl-	—	－	0.39
VOA16	Cyclohexyl-2，4-dimethylbenzene ketone	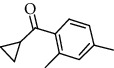	C_15_H_20_O	0.15
VOA17	*α*-Gurjunene	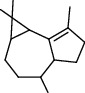	C_15_H_24_	0.16
VOA18	Shyobunone	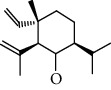	C_15_H_24_O	0.80
VOA19	1,6,10-Dodecatrien-3-ol, 3,7,11-trimethyl-	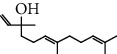	C_15_H_26_O	0.22
VOA20	Benzene, 1,2,3-trimethoxy-5-(2-propenyl)-	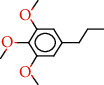	C_15_H_24_O	4.84
VOA21	Hexadecane	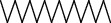	C_16_H_34_	0.08
VOA22	Tumerone	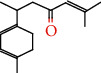	C_15_H_22_O	0.23
VOA23	*β*-Asarone	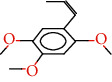	C_12_H_16_O_3_	71.31
VOA24	Cyclohexanone, 6-furfurylidene-2,2,3-trimethyl-	—	－	0.25
VOA25	Naphthalene, 1,2,3,5,6,7,8,8a-octahydro-1,8a-dimethyl-7-(1-methylethenyl)-, [1R-(1.alpha.,7.beta.,8a.alpha.)]-	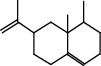	C_15_H_24_	0.33
VOA26	Spiro[4.5]dec-6-en-8-one, 1,7-dimethyl-4-(1-methylethyl)-	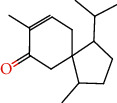	C_15_H_24_O	0.45
VOA27	*α*-Asarone	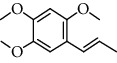	C_12_H_16_O_3_	7.26
VOA28	2H-Cyclopropa[a]naphthalen-2-one, 1,1a,4,5,6,7,7a,7b-octahydro-1,1,7,7a-tetramethyl-, (1a.alpha.,7.alpha.,7a.alpha.,7b.alpha.)-	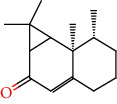	C_15_H_22_O	2.45
VOA29	Cyclolongifolene oxide, dehydro-	—	C_15_H_22_O	0.29
VOA30	2,4,6-Octatriene, 2,6-dimethyl-	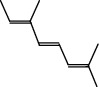	C_10_H_16_	1.39
VOA31	Bicyclo[3.1.1]hept-3-en-2-one, 4,6,6-trimethyl-, (1S)-	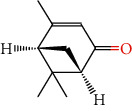	C_10_H_14_O	0.22
VOA32	Isocalamendiol	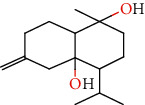	C_15_H_26_O_2_	0.38
VOA33	5(1H)-Azulenone, 2,4,6,7,8,8a-hexahydro-3,8-dimethyl-4-(1-methylethylidene)-, (8S-cis)-	—	C_15_H_22_O	0.14

**Table 2:** Molecular docking of 5 important targets of VOA.

Targets	PDB ID	Chemical composition	Docking score
ESR1	6PET	VOA1	64.5085
P2RX7	5U2H	VOA1	62.8344
PTGS2	5KIR	VOA1	63.5080
ESR1	6PET	VOA2	67.2673
P2RX7	5U2H	VOA2	60.5152
PTGS2	5KIR	VOA2	62.7291
ESR1	6PET	VOA3	64.0894
P2RX7	5U2H	VOA3	62.1425
PTGS2	5KIR	VOA3	64.5139
ESR1	6PET	VOA4	67.2681
PPAR-*α*	3FEI	VOA4	68.7501
P2RX7	5U2H	VOA4	66.2316
PTGS2	5KIR	VOA4	62.2260
EPHX2	3ANS	VOA4	62.6863
ESR1	6PET	VOA5	66.2105
PPAR-*α*	3FEI	VOA5	65.4939
P2RX7	5U2H	VOA5	67.7889
PTGS2	5KIR	VOA5	64.5092
EPHX2	3ANS	VOA5	65.3003
ESR1	6PET	VOA6	65.2575
PPAR-*α*	3FEI	VOA6	67.6596
P2RX7	5U2H	VOA6	66.9050
PTGS2	5KIR	VOA6	63.6252
EPHX2	3ANS	VOA6	65.4785
ESR1	6PET	VOA7	60.2374
PTGS2	5KIR	VOA7	64.371
EPHX2	3ANS	VOA7	61.3399
ESR1	6PET	VOA8	67.4526
PTGS2	5KIR	VOA8	64.3934
EPHX2	3ANS	VOA8	61.9769
ESR1	6PET	VOA9	68.2695
PPAR-*α*	3FEI	VOA9	71.5129
P2RX7	5U2H	VOA9	72.5833
PTGS2	5KIR	VOA9	71.6689
EPHX2	3ANS	VOA9	71.4170
ESR1	6PET	VOA11	78.1423
PPAR-*α*	3FEI	VOA11	66.9132
PTGS2	5KIR	VOA11	77.6608
EPHX2	3ANS	VOA11	70.2722
ESR1	6PET	VOA12	80.9465
PPAR-*α*	3FEI	VOA12	74.2676
P2RX7	5U2H	VOA12	90.9012
PTGS2	5KIR	VOA12	77.8928
EPHX2	3ANS	VOA12	77.0346
ESR1	6PET	VOA15	78.0546
PPAR-*α*	3FEI	VOA15	76.7342
P2RX7	5U2H	VOA15	83.8674
PTGS2	5KIR	VOA15	79.1801
EPHX2	3ANS	VOA15	74.6761
ESR1	6PET	VOA16	85.9312
PPAR-*α*	3FEI	VOA16	83.2731
P2RX7	5U2H	VOA16	62.2699
PTGS2	5KIR	VOA16	92.4880
EPHX2	3ANS	VOA16	89.1537
ESR1	6PET	VOA18	83.9547
PPAR-*α*	3FEI	VOA18	65.3407
PTGS2	5KIR	VOA18	85.8182
EPHX2	3ANS	VOA18	87.7297
ESR1	6PET	VOA20	96.4940
PPAR-*α*	3FEI	VOA20	86.9967
P2RX7	5U2H	VOA20	97.3168
PTGS2	5KIR	VOA20	104.678
EPHX2	3ANS	VOA20	93.5532
ESR1	6PET	VOA21	67.9247
PPAR-*α*	3FEI	VOA21	65.9274
PTGS2	5KIR	VOA21	66.2899
EPHX2	3ANS	VOA21	67.4975
ESR1	6PET	VOA22	69.3249
PPAR-*α*	3FEI	VOA22	73.3223
P2RX7	5U2H	VOA22	65.2278
PTGS2	5KIR	VOA22	70.7295
EPHX2	3ANS	VOA22	74.0924
ESR1	6PET	VOA23	96.9734
PPAR-*α*	3FEI	VOA23	99.8045
P2RX7	5U2H	VOA23	87.1190
PTGS2	5KIR	VOA23	102.692
EPHX2	3ANS	VOA23	88.7468
ESR1	6PET	VOA25	74.0667
PPAR-*α*	3FEI	VOA25	64.8149
PTGS2	5KIR	VOA25	83.2913
EPHX2	3ANS	VOA25	77.1942
ESR1	6PET	VOA26	62.8586
PTGS2	5KIR	VOA26	90.0276
EPHX2	3ANS	VOA26	90.5615
ESR1	6PET	VOA27	84.2810
PPAR-*α*	3FEI	VOA27	92.7826
P2RX7	5U2H	VOA27	81.9188
PTGS2	5KIR	VOA27	101.013
EPHX2	3ANS	VOA27	83.5758
PTGS2	5KIR	VOA29	81.0304
EPHX2	3ANS	VOA29	81.9996
ESR1	6PET	VOA30	65.5115
PPAR-*α*	3FEI	VOA30	62.0720
P2RX7	5U2H	VOA30	66.2747
PTGS2	5KIR	VOA30	67.1910
EPHX2	3ANS	VOA30	63.4045
ESR1	6PET	VOA31	62.1971
P2RX7	5U2H	VOA31	64.2998
EPHX2	3ANS	VOA31	66.0117
PPAR-*α*	3FEI	VOA32	61.9083
PTGS2	5KIR	VOA32	84.2783
EPHX2	3ANS	VOA32	80.7550
ESR1	6PET	Propranolol	95.8670
PPAR-*α*	3FEI	Propranolol	98.2171
P2RX7	5U2H	Propranolol	90.1426
PTGS2	5KIR	Propranolol	112.0720
EPHX2	3ANS	Propranolol	107.5180

## Data Availability

All datasets used to support the findings of this study are included within this study/supplementary materials.

## Supplementary Materials

Supplementary Materials Detailed search strategy.
