# Supplementary material for: Uncovering the Protective Mechanism of the Volatile Oil of Acorus tatarinowii against Acute Myocardial Ischemia Injury Using Network Pharmacology and Experimental Validation
Source: Evid Based Complement Alternat Med. 2021 Jun 22;2021:6630795. doi: 10.1155/2021/6630795 (PMC8241509; doi:10.1155/2021/6630795)
Supplement: Supplementary Materials — Detailed search strategy. [file 6630795.f1.zip › 6630795.f1/Supplementary Table S1.docx]

Table S1 The detail information of Component-related targets

| Number | Component-related targets |
| --- | --- |
| 1 | AHR |
| 2 | AR |
| 3 | ATAD5 |
| 4 | ATF6 |
| 5 | BCHE |
| 6 | CASP3 |
| 7 | CASP7 |
| 8 | CER1 |
| 9 | CHRM2 |
| 10 | CHRNA2 |
| 11 | CNR2 |
| 12 | CYP19A1 |
| 13 | CYP2C19 |
| 14 | ESR1 |
| 15 | ESR2 |
| 16 | ESRRA |
| 17 | FAAH |
| 18 | GABRA1 |
| 19 | GABRA2 |
| 20 | GABRA5 |
| 21 | GABRA6 |
| 22 | GLI3 |
| 23 | HDAC9 |
| 24 | HIF1A |
| 25 | HSPB1 |
| 26 | JUN |
| 27 | MAPK11 |
| 28 | MAPK12 |
| 29 | MAPK13 |
| 30 | MAPK14 |
| 31 | NFE2L2 |
| 32 | NFKB1 |
| 33 | NR1H4 |
| 34 | NR1I2 |
| 35 | NR1I3 |
| 36 | NR3C1 |
| 37 | PGR |
| 38 | PPARA |
| 39 | PPARD |
| 40 | PPARG |
| 41 | PRKACA |
| 42 | PTGS2 |
| 43 | PTPN1 |
| 44 | RARA |
| 45 | RXRA |
| 46 | SLC6A2 |
| 47 | SLC6A4 |
| 48 | SMAD2 |
| 49 | SMAD3 |
| 50 | TP53 |
| 51 | TRHR |
| 52 | TRPV1 |
| 53 | TSHR |
| 54 | VDR |
| 55 | ADRA1B |
| 56 | CHRM1 |
| 57 | CHRM3 |
| 58 | MAOB |
| 59 | NCOA2 |
| 60 | PTGS1 |
| 61 | RXRG |
| 62 | LYZL6 |
| 63 | TAAR1 |
| 64 | ADH1B |
| 65 | ADH1C |
| 66 | GABRA3 |
| 67 | IGHG1 |
| 68 | IPP |
| 69 | MTRR |
| 70 | NCOA1 |
| 71 | NOS1 |
| 72 | NOS2 |
| 73 | NOS3 |
| 74 | NR1H3 |
| 75 | POR |
| 76 | ADH1A |
| 77 | DPP4 |
| 78 | GLI1 |
| 79 | GLI2 |
| 80 | PLK1 |
| 81 | ABCB6 |
| 82 | ABHD5 |
| 83 | ABL1 |
| 84 | ACP1 |
| 85 | ADAM10 |
| 86 | ADAM17 |
| 87 | ADRB2 |
| 88 | AKR1B10 |
| 89 | AKT1 |
| 90 | ALDH1A1 |
| 91 | ALOX12 |
| 92 | ALOX15 |
| 93 | ALOX15B |
| 94 | ALPI |
| 95 | APAF1 |
| 96 | APLNR |
| 97 | APOBEC3A |
| 98 | APOBEC3F |
| 99 | APOBEC3G |
| 100 | ARNT |
| 101 | ARRB1 |
| 102 | ASAP1 |
| 103 | ATG4B |
| 104 | ATM |
| 105 | ATXN2 |
| 106 | AVPR1A |
| 107 | BAZ2B |
| 108 | BCL2L1 |
| 109 | BCL2L11 |
| 110 | BRCA1 |
| 111 | CA1 |
| 112 | CA2 |
| 113 | CA4 |
| 114 | CASP1 |
| 115 | CASP6 |
| 116 | caspase7 |
| 117 | cathepsinL1(human) |
| 118 | CBX1 |
| 119 | CCR6 |
| 120 | CCT2 |
| 121 | CDC25A |
| 122 | CDK5 |
| 123 | CDK5R1 |
| 124 | CES1 |
| 125 | CES2 |
| 126 | CFTR |
| 127 | CGA |
| 128 | CGAS |
| 129 | ChainA |
| 130 | ChainB |
| 131 | ChainE |
| 132 | CHRM4 |
| 133 | CHRM5 |
| 134 | COPS5 |
| 135 | CRHBP |
| 136 | CRHR2 |
| 137 | CTDSP1 |
| 138 | CTSK |
| 139 | CXCL8 |
| 140 | CXCR6 |
| 141 | CYP3A4 |
| 142 | DAGLB |
| 143 | DNMT1 |
| 144 | DRD1 |
| 145 | DRD2 |
| 146 | DRD3 |
| 147 | EPAS1 |
| 148 | ERAP1 |
| 149 | ERG |
| 150 | euchromatichistone-lysineN-methyltransferase2 |
| 151 | EYA2 |
| 152 | FASN |
| 153 | FEN1 |
| 154 | FGF22 |
| 155 | Fosb |
| 156 | FXN |
| 157 | G6PD |
| 158 | GAA |
| 159 | GABBR1 |
| 160 | GALR3 |
| 161 | GBA |
| 162 | GLA |
| 163 | GLP1R |
| 164 | GLS |
| 165 | GMNN |
| 166 | GNAI1 |
| 167 | GNAS |
| 168 | GOPC |
| 169 | GPBAR1 |
| 170 | GPR183 |
| 171 | GRK2 |
| 172 | GSK3A |
| 173 | GSTO1 |
| 174 | HBB |
| 175 | HCRTR1 |
| 176 | HDAC3 |
| 177 | HKDC1 |
| 178 | HOXA9 |
| 179 | HPGD |
| 180 | HSD17B3 |
| 181 | HTR5A |
| 182 | HTRA1 |
| 183 | HTT |
| 184 | IDE |
| 185 | IDH1 |
| 186 | IL1B |
| 187 | ITGA4 |
| 188 | KAT2A |
| 189 | KCNH2 |
| 190 | KCNK3 |
| 191 | KEAP1 |
| 192 | KLK7 |
| 193 | KPNB1 |
| 194 | L3MBTL1 |
| 195 | LARGE1 |
| 196 | LYPLA1 |
| 197 | LYPLA2 |
| 198 | MAPK1 |
| 199 | MAPT |
| 200 | MARVELD2 |
| 201 | MBD2 |
| 202 | MC4R |
| 203 | MCL1 |
| 204 | MCOLN1 |
| 205 | MDM2 |
| 206 | MDM4 |
| 207 | MITF |
| 208 | MLLT3 |
| 209 | MMP14 |
| 210 | MRGPRX1 |
| 211 | NAE1 |
| 212 | NCOA3 |
| 213 | NFKBIA |
| 214 | NLRP3 |
| 215 | NOD2 |
| 216 | NPC1 |
| 217 | NPC1L1 |
| 218 | NR0B1 |
| 219 | NR2E3 |
| 220 | NR2F2 |
| 221 | NR5A2 |
| 222 | NTSR1 |
| 223 | OPRD1 |
| 224 | OPRM1 |
| 225 | OXTR |
| 226 | PABPC1 |
| 227 | PADI4 |
| 228 | PAFAH1B2 |
| 229 | PAFAH1B3 |
| 230 | PAFAH2 |
| 231 | PDIA6 |
| 232 | PI4K2A |
| 233 | PIN1 |
| 234 | PINK1 |
| 235 | PIP4K2A |
| 236 | PLA2G3 |
| 237 | PLA2G7 |
| 238 | PLCB3 |
| 239 | PLCG1 |
| 240 | PLIN5 |
| 241 | POLB |
| 242 | POLH |
| 243 | POLI |
| 244 | POLK |
| 245 | PPME1 |
| 246 | PPP1CA |
| 247 | PPP5C |
| 248 | PREPL |
| 249 | PRKACB |
| 250 | PRKAR2B |
| 251 | PRKN |
| 252 | PRMT1 |
| 253 | PRNP |
| 254 | PSIP1 |
| 255 | PSMD14 |
| 256 | PTH1R |
| 257 | PTPN5 |
| 258 | RAB9A |
| 259 | RACGAP1 |
| 260 | RAD52 |
| 261 | RAD54L |
| 262 | RAN |
| 263 | RAPGEF3 |
| 264 | RAPGEF4 |
| 265 | RBBP8 |
| 266 | RGS12 |
| 267 | RGS4 |
| 268 | RIN1 |
| 269 | RIPK2 |
| 270 | RORC |
| 271 | RXFP1 |
| 272 | S100A4 |
| 273 | SENP6 |
| 274 | SENP7 |
| 275 | SENP8 |
| 276 | sentrin |
| 277 | SERPINA6 |
| 278 | SHBG |
| 279 | SIAE |
| 280 | SIRT5 |
| 281 | SIX1 |
| 282 | SLC5A7 |
| 283 | SMARCA2 |
| 284 | SMN2 |
| 285 | SMPD1 |
| 286 | SNCA |
| 287 | SNUPN |
| 288 | STK33 |
| 289 | SUMO1 |
| 290 | Tcellreceptor,partial |
| 291 | TACC3 |
| 292 | TARDBP |
| 293 | TDP1 |
| 294 | TDP2 |
| 295 | THRB |
| 296 | TLR9 |
| 297 | TNF |
| 298 | TNFRSF10B |
| 299 | TNNC1 |
| 300 | TNNI3 |
| 301 | TNNT2 |
| 302 | TRA |
| 303 | TSG101 |
| 304 | UBE2M |
| 305 | UBE2N |
| 306 | UCHL5 |
| 307 | UHRF1 |
| 308 | USP1 |
| 309 | USP2 |
| 310 | WRN |
| 311 | ADRA1A |
| 312 | CDC25B |
| 313 | CYP51A1 |
| 314 | HSD11B1 |
| 315 | POLA1 |
| 316 | SHH |
| 317 | SIGMAR1 |
| 318 | TRPM8 |
| 319 | UGT1A1 |
| 320 | UGT1A4 |
| 321 | UGT2B7 |
| 322 | XRCC2 |
| 323 | ABAT |
| 324 | ADRA1D |
| 325 | ADRA2A |
| 326 | ADRA2C |
| 327 | ADRB1 |
| 328 | ALDH5A1 |
| 329 | BLM |
| 330 | BRD9 |
| 331 | CA5A |
| 332 | caspase7,apoptosis-relatedcysteinepeptidase |
| 333 | CTSL |
| 334 | COA7 |
| 335 | CTRB1 |
| 336 | CYP1A2 |
| 337 | CYP2A6 |
| 338 | CYP2E1 |
| 339 | DRD4 |
| 340 | GABRB3 |
| 341 | GAPDH |
| 342 | heatshock70kDaprotein5 |
| 343 | HTRA1protein |
| 344 | KAT2B |
| 345 | KCNK2 |
| 346 | KMT2A |
| 347 | MAOA |
| 348 | MEN1 |
| 349 | MPO |
| 350 | NQO1 |
| 351 | NQO2 |
| 352 | P2RX7 |
| 353 | PDE7A |
| 354 | PRSS3 |
| 355 | PTGER2 |
| 356 | SENP1 |
| 357 | SLC6A3 |
| 358 | SPICE1 |
| 359 | SRD5A1 |
| 360 | TTR |
| 361 | SLCO1B1 |
| 362 | SLCO1B3 |
| 363 | ABCB1 |
| 364 | ADRA2B |
| 365 | ALOX5 |
| 366 | APP |
| 367 | BDKRB2 |
| 368 | BRPF1 |
| 369 | CA12 |
| 370 | CA13 |
| 371 | CA14 |
| 372 | CA3 |
| 373 | CA5B |
| 374 | CA6 |
| 375 | CA7 |
| 376 | CA9 |
| 377 | CDC7 |
| 378 | CDK2 |
| 379 | CYP11B1 |
| 380 | CYP11B2 |
| 381 | CYP17A1 |
| 382 | CYP1A1 |
| 383 | CYP1B1 |
| 384 | CYP2C9 |
| 385 | EGFR |
| 386 | GRM2 |
| 387 | GRM5 |
| 388 | GSK3B |
| 389 | HDAC8 |
| 390 | HMGCR |
| 391 | HTR2A |
| 392 | HTR6 |
| 393 | IDO1 |
| 394 | JAK2 |
| 395 | LCK |
| 396 | LTA4H |
| 397 | MIF |
| 398 | MKNK1 |
| 399 | NUDT1 |
| 400 | PDE10A |
| 401 | PIK3CA |
| 402 | PIK3CB |
| 403 | PRKCA |
| 404 | RELA |
| 405 | SCN5A |
| 406 | STAT3 |
| 407 | TBXAS1 |
| 408 | TMIGD3 |
| 409 | TUBB1 |
| 410 | TUBB3 |
| 411 | TYK2 |
| 412 | XPO1 |
| 413 | ADORA1 |
| 414 | ADORA2A |
| 415 | ADORA3 |
| 416 | SRD5A2 |
| 417 | HTR2C |
| 418 | HAO1 |
| 419 | MGLL |
| 420 | PREP |
| 421 | C5AR1 |
| 422 | HDAC6 |
| 423 | NR1H2 |
| 424 | CNR1 |
| 425 | THRA |
| 426 | EPHX2 |
| 427 | CACNA1B |
| 428 | CACNA2D1 |
| 429 | G6PC |
| 430 | GRM1 |
| 431 | TSPO |
| 432 | KCNN4 |
| 433 | KCNA3 |
| 434 | AKR1C3 |
| 435 | CCR5 |
| 436 | OPRL1 |
| 437 | PTGDR2 |
| 438 | TYMS |
| 439 | GABRG2 |
| 440 | DNMT3A |
| 441 | CRHR1 |
| 442 | HCRTR2 |
| 443 | HSP90AA1 |
| 444 | PDE3A |
| 445 | CHRNA7 |
| 446 | CTSB |
| 447 | EPHX1 |
| 448 | MTNR1A |
| 449 | MTNR1B |
| 450 | PARP1 |
| 451 | PTGES |
| 452 | FABP1 |
| 453 | HTR7 |
| 454 | NR3C2 |
| 455 | PTPN2 |
| 456 | TRPA1 |
| 457 | ABHD6 |
| 458 | BACE1 |
| 459 | BRS3 |
| 460 | CASR |
| 461 | CCR1 |
| 462 | CCR9 |
| 463 | F2R |
| 464 | FDFT1 |
| 465 | FNTA |
| 466 | GABBR2 |
| 467 | GCGR |
| 468 | GCK |
| 469 | GLUL |
| 470 | HRH3 |
| 471 | HSD11B2 |
| 472 | HSD17B2 |
| 473 | ICMT |
| 474 | IL6ST |
| 475 | JAK1 |
| 476 | JAK3 |
| 477 | KCNA5 |
| 478 | LIPE |
| 479 | MAPK8 |
| 480 | NPY2R |
| 481 | NPY5R |
| 482 | PDE2A |
| 483 | PER2 |
| 484 | PGGT1B |
| 485 | PRCP |
| 486 | PRKCB |
| 487 | PRKCD |
| 488 | PRKCE |
| 489 | PRKCG |
| 490 | PRKCH |
| 491 | PRKCQ |
| 492 | PSEN2 |
| 493 | RASGRP1 |
| 494 | SCN9A |
| 495 | SLC10A2 |
| 496 | SQLE |
| 497 | TNK2 |
| 498 | TTL |
| 499 | AVPR2 |
| 500 | CETP |
| 501 | CTSS |
| 502 | DUSP3 |
| 503 | GRK6 |
| 504 | GRM4 |
| 505 | HTR2B |
| 506 | IGF1R |
| 507 | MAPK10 |
| 508 | PDE4A |
| 509 | PDE4B |
| 510 | PTGFR |
| 511 | PTK2 |
| 512 | RAD1 |
| 513 | RAD51 |
| 514 | RORA |
| 515 | RORB |
| 516 | SLC18A2 |
| 517 | SLC6A9 |
| 518 | TACR2 |
| 519 | TACR3 |
| 520 | TGFBR1 |
| 521 | TYR |
| 522 | VCP |
| 523 | DNM1 |
| 524 | LPL |
| 525 | BRD4 |
| 526 | CHRNA3 |
| 527 | DRD5 |
| 528 | FCGR1A |
| 529 | HDAC1 |
| 530 | HRH1 |
| 531 | HRH2 |
| 532 | HTR1A |
| 533 | HTR1B |
| 534 | HTR1D |
| 535 | HTR1E |
| 536 | HTR3A |
| 537 | MMP13 |
| 538 | S1PR3 |
| 539 | SIRT2 |
| 540 | TACR1 |
| 541 | ABCG2 |
| 542 | ADORA2B |
| 543 | CCNB3 |
| 544 | CCND3 |
| 545 | CCNE2 |
| 546 | CDK1 |
| 547 | CDK7 |
| 548 | CSNK1D |
| 549 | CSNK1E |
| 550 | ELANE |
| 551 | FOS |
| 552 | GAD2 |
| 553 | KDR |
| 554 | MAP3K14 |
| 555 | MET |
| 556 | MST1R |
| 557 | PDE4D |
| 558 | PKIA |
| 559 | TNKS |
| 560 | TNKS2 |
| 561 | BRD2 |
| 562 | CTSC |
| 563 | CTSF |
| 564 | CTSV |
| 565 | IMPDH2 |
| 566 | CTSD |
| 567 | MAPK3 |
| 568 | PTPN11 |
| 569 | CYP2D6 |
| 570 | PTPN6 |
| 571 | CYP2B6 |
| 572 | FFAR1 |
| 573 | FFAR4 |
| 574 | ACACB |
| 575 | ABCC9 |
| 576 | AOC3 |
| 577 | CXCR2 |
| 578 | FKBP1A |
| 579 | HMOX1 |
| 580 | KCNE1 |
| 581 | KCNJ11 |
| 582 | LRRK2 |
| 583 | MMP1 |
| 584 | PIM1 |
| 585 | PIM3 |
| 586 | PYGL |
| 587 | TRPV3 |
| 588 | MPI |
| 589 | ACHE |
