# Supplementary material for: Uncovering the Protective Mechanism of the Volatile Oil of Acorus tatarinowii against Acute Myocardial Ischemia Injury Using Network Pharmacology and Experimental Validation
Source: Evid Based Complement Alternat Med. 2021 Jun 22;2021:6630795. doi: 10.1155/2021/6630795 (PMC8241509; doi:10.1155/2021/6630795)
Supplement: Supplementary Materials — Detailed search strategy. [file 6630795.f1.zip › 6630795.f1/Supplementary Table S2.docx]

Table S2 The detail information of disease related targets

| Number | Disease related targets |
| --- | --- |
| 1 | CCL2 |
| 2 | EDN1 |
| 3 | KCNJ8 |
| 4 | NOS3 |
| 5 | CRP |
| 6 | AGT |
| 7 | VEGFA |
| 8 | HGF |
| 9 | IGF1 |
| 10 | IL6 |
| 11 | TNF |
| 12 | IL1B |
| 13 | ICAM1 |
| 14 | HMGB1 |
| 15 | ADRB2 |
| 16 | MAPK14 |
| 17 | HMOX1 |
| 18 | IGFBP3 |
| 19 | CRK |
| 20 | GCLC |
| 21 | F3 |
| 22 | SOD2 |
| 23 | FGF1 |
| 24 | IL1A |
| 25 | ELN |
| 26 | ADIPOQ |
| 27 | FGF2 |
| 28 | JAK2 |
| 29 | SELP |
| 30 | SLC6A6 |
| 31 | POMC |
| 32 | ADRB1 |
| 33 | PGM1 |
| 34 | FABP5 |
| 35 | PDE4B |
| 36 | PFKM |
| 37 | PFKFB3 |
| 38 | F2RL1 |
| 39 | PDK4 |
| 40 | PDHA1 |
| 41 | ENSA |
| 42 | RXRG |
| 43 | RTN1 |
| 44 | RPL17 |
| 45 | DUSP1 |
| 46 | DUSP6 |
| 47 | RAB5A |
| 48 | PYGB |
| 49 | PTPRA |
| 50 | PSMB5 |
| 51 | PRKCE |
| 52 | PPP3R1 |
| 53 | PPP2CA |
| 54 | EGR2 |
| 55 | PPM1B |
| 56 | PPAT |
| 57 | DLAT |
| 58 | PEBP1 |
| 59 | P4HB |
| 60 | HIVEP2 |
| 61 | HK1 |
| 62 | HK2 |
| 63 | MX1 |
| 64 | HMGCS2 |
| 65 | NR4A1 |
| 66 | KDR |
| 67 | HSPA1A |
| 68 | HSPA2 |
| 69 | JUNB |
| 70 | HSPA9 |
| 71 | IRF1 |
| 72 | ID1 |
| 73 | ID3 |
| 74 | IFNGR1 |
| 75 | CXCL10 |
| 76 | MEOX2 |
| 77 | HADHA |
| 78 | GYG1 |
| 79 | NPPA |
| 80 | GATM |
| 81 | GBP2 |
| 82 | GCH1 |
| 83 | NFKBIA |
| 84 | NEDD4 |
| 85 | GHR |
| 86 | NCL |
| 87 | GK |
| 88 | GNA12 |
| 89 | ALDH6A1 |
| 90 | CXCL2 |
| 91 | GSTA1 |
| 92 | GSTM2 |
| 93 | GSTP1 |
| 94 | KITLG |
| 95 | IL6ST |
| 96 | CANX |
| 97 | EIF2AK3 |
| 98 | GDF15 |
| 99 | PRDX6 |
| 100 | APLP2 |
| 101 | FEZ2 |
| 102 | DNM1L |
| 103 | CLEC10A |
| 104 | PDLIM5 |
| 105 | KLF4 |
| 106 | AREG |
| 107 | CCND1 |
| 108 | RAB7A |
| 109 | BHLHE40 |
| 110 | ATP1A1 |
| 111 | SNAP23 |
| 112 | SOCS3 |
| 113 | ARNTL |
| 114 | RHOC |
| 115 | RALBP1 |
| 116 | ALDOA |
| 117 | ADM2 |
| 118 | RAB1B |
| 119 | EIF2A |
| 120 | CTTNBP2 |
| 121 | RSAD2 |
| 122 | RLN3 |
| 123 | ACTB |
| 124 | RAB12 |
| 125 | GAS5 |
| 126 | FEV |
| 127 | TMED2 |
| 128 | ACOT2 |
| 129 | MAPRE1 |
| 130 | AK1 |
| 131 | CES3 |
| 132 | NPTN |
| 133 | RGCC |
| 134 | AES |
| 135 | TUBA1A |
| 136 | ZFP36 |
| 137 | ZEB1 |
| 138 | TMPO |
| 139 | SELE |
| 140 | SLC8A1 |
| 141 | CDH2 |
| 142 | CPT1B |
| 143 | SPARC |
| 144 | CEBPD |
| 145 | TLE4 |
| 146 | COX5B |
| 147 | CTSC |
| 148 | TFRC |
| 149 | MAP3K8 |
| 150 | TAP1 |
| 151 | CIRBP |
| 152 | STAT5A |
| 153 | CD38 |
| 154 | CCL4 |
| 155 | C1QB |
| 156 | RYR2 |
| 157 | VCAM1 |
| 158 | UQCRFS1 |
| 159 | CALR |
| 160 | CX3CL1 |
| 161 | CAMK2D |
| 162 | PLA2G2A |
| 163 | MSN |
| 164 | UGCG |
| 165 | TXNRD1 |
| 166 | DCN |
| 167 | TNNT2 |
| 168 | CCND2 |
| 169 | CCL3 |
| 170 | CD36 |
| 171 | SULT1A1 |
| 172 | MMP9 |
| 173 | CCR5 |
| 174 | LOX |
| 175 | CD40LG |
| 176 | ABCC9 |
| 177 | HLA-E |
| 178 | ACE |
| 179 | LPL |
| 180 | APOB |
| 181 | APOE |
| 182 | HIF1A |
| 183 | MTHFR |
| 184 | ABCA1 |
| 185 | VWF |
| 186 | AGTR1 |
| 187 | ESR1 |
| 188 | F7 |
| 189 | APOA1 |
| 190 | ANGPT1 |
| 191 | CETP |
| 192 | ALDH2 |
| 193 | LPA |
| 194 | LDLR |
| 195 | CTGF |
| 196 | KLK1 |
| 197 | F2 |
| 198 | SERPINE1 |
| 199 | FGB |
| 200 | F5 |
| 201 | HSPA4 |
| 202 | MBL2 |
| 203 | AKR1B1 |
| 204 | PPARA |
| 205 | PON1 |
| 206 | APLNR |
| 207 | REN |
| 208 | SCG2 |
| 209 | MTTP |
| 210 | NCAM1 |
| 211 | PGR-AS1 |
| 212 | FSD1 |
| 213 | VEGFB |
| 214 | SOD3 |
| 215 | ACE2 |
| 216 | FGF4 |
| 217 | APOC3 |
| 218 | CYP2C9 |
| 219 | AMPD1 |
| 220 | CYP2D6 |
| 221 | TXNIP |
| 222 | EEF1A2 |
| 223 | MAPK3 |
| 224 | EDNRB |
| 225 | PARP1 |
| 226 | SESN2 |
| 227 | CHIT1 |
| 228 | IL10 |
| 229 | TLR4 |
| 230 | PCSK9 |
| 231 | ZNF202 |
| 232 | ATM |
| 233 | ENTPD1 |
| 234 | CD14 |
| 235 | HSPB1 |
| 236 | BCL2 |
| 237 | RUNX1 |
| 238 | IL1RN |
| 239 | HSPB3 |
| 240 | FSD1L |
| 241 | HSPB2 |
| 242 | SLC33A1 |
| 243 | IL2 |
| 244 | ANXA5 |
| 245 | VEGFC |
| 246 | SLC23A1 |
| 247 | ABCG1 |
| 248 | ANK2 |
| 249 | TRIB1 |
| 250 | CAD |
| 251 | IL18BP |
| 252 | EDIL3 |
| 253 | YAP1 |
| 254 | APRT |
| 255 | NOD1 |
| 256 | BMP2 |
| 257 | AIRE |
| 258 | KLK4 |
| 259 | NPHS2 |
| 260 | LONP1 |
| 261 | PPIG |
| 262 | ABCC6 |
| 263 | PER2 |
| 264 | SPHK1 |
| 265 | ATF3 |
| 266 | S1PR2 |
| 267 | MSC |
| 268 | KL |
| 269 | AQP4 |
| 270 | DYSF |
| 271 | GMFG |
| 272 | MANF |
| 273 | ATP5PF |
| 274 | PLA2G6 |
| 275 | ATP2B1 |
| 276 | MIR141 |
| 277 | CREG1 |
| 278 | XPR1 |
| 279 | ABRAXAS2 |
| 280 | ABO |
| 281 | SLC35G1 |
| 282 | LYPD4 |
| 283 | RBM45 |
| 284 | HSPB6 |
| 285 | CYP2R1 |
| 286 | ADH1B |
| 287 | ADH1C |
| 288 | TRIM63 |
| 289 | ADORA1 |
| 290 | ADORA2B |
| 291 | UTS2B |
| 292 | COPD |
| 293 | IRF2BP2 |
| 294 | CCR2 |
| 295 | MIR451A |
| 296 | MIR377 |
| 297 | TRIM72 |
| 298 | MIR151A |
| 299 | MIR214 |
| 300 | MIR210 |
| 301 | MIR21 |
| 302 | MIR150 |
| 303 | MIR145 |
| 304 | MIR144 |
| 305 | RNF146 |
| 306 | ZC3H12A |
| 307 | PARP4 |
| 308 | SIRT1 |
| 309 | ARC |
| 310 | SETD2 |
| 311 | GPD1L |
| 312 | AKT2 |
| 313 | ADAMTS13 |
| 314 | KCNQ1OT1 |
| 315 | ALB |
| 316 | UTS2 |
| 317 | NMU |
| 318 | CYSLTR1 |
| 319 | PADI4 |
| 320 | PPP1R15A |
| 321 | TIPARP |
| 322 | ADRB3 |
| 323 | MAGEE1 |
| 324 | GRK2 |
| 325 | UGT1A1 |
| 326 | UGT1A6 |
| 327 | ISYNA1 |
| 328 | NPC1L1 |
| 329 | SENP1 |
| 330 | AGER |
| 331 | TOR2A |
| 332 | ANKRD1 |
| 333 | HYOU1 |
| 334 | CALM1 |
| 335 | PLG |
| 336 | NPPC |
| 337 | NPPB |
| 338 | FHL2 |
| 339 | FOXO1 |
| 340 | NOS2 |
| 341 | GCKR |
| 342 | GDF10 |
| 343 | GJA1 |
| 344 | GJA4 |
| 345 | GCLM |
| 346 | COX2 |
| 347 | MSX2 |
| 348 | NPR1 |
| 349 | OLR1 |
| 350 | PLD1 |
| 351 | EPRS |
| 352 | PIK3CG |
| 353 | PIK3CD |
| 354 | PIK3CB |
| 355 | PIK3CA |
| 356 | ERBB2 |
| 357 | PECAM1 |
| 358 | FABP3 |
| 359 | SERPINB2 |
| 360 | P2RX7 |
| 361 | TNFRSF11B |
| 362 | UTS2R |
| 363 | MPO |
| 364 | KIT |
| 365 | KISS1 |
| 366 | HRH2 |
| 367 | KCNH2 |
| 368 | HSPA5 |
| 369 | INS |
| 370 | IFRD1 |
| 371 | IL18 |
| 372 | CXCL8 |
| 373 | IGF2 |
| 374 | IGFBP1 |
| 375 | IGFBP7 |
| 376 | KNG1 |
| 377 | LCT |
| 378 | GRK5 |
| 379 | MMP14 |
| 380 | GSTM1 |
| 381 | MMP2 |
| 382 | MIF |
| 383 | GSTT1 |
| 384 | HBA1 |
| 385 | LTC4S |
| 386 | LTA |
| 387 | HFE |
| 388 | HLA-DRB1 |
| 389 | LDHA |
| 390 | CYR61 |
| 391 | FXYD1 |
| 392 | CALM2 |
| 393 | CHRNA4 |
| 394 | ABCC8 |
| 395 | COL5A2 |
| 396 | STAT6 |
| 397 | COMT |
| 398 | STAT3 |
| 399 | SPP1 |
| 400 | COX8A |
| 401 | SLN |
| 402 | CYBA |
| 403 | CYP2B6 |
| 404 | SLC5A1 |
| 405 | TCF7L2 |
| 406 | TDGF1 |
| 407 | CALM3 |
| 408 | CAV3 |
| 409 | UCN |
| 410 | TTN |
| 411 | CCNA2 |
| 412 | TNFRSF1B |
| 413 | TNFRSF1A |
| 414 | TLR2 |
| 415 | THPO |
| 416 | TGFB1 |
| 417 | CHI3L1 |
| 418 | TFPI |
| 419 | SHH |
| 420 | CYP2C19 |
| 421 | EDNRA |
| 422 | PTMS |
| 423 | PTGS2 |
| 424 | PRKAB1 |
| 425 | PRKAA2 |
| 426 | PRKAA1 |
| 427 | EEF2 |
| 428 | PPP3CA |
| 429 | EGR1 |
| 430 | EIF5A |
| 431 | ENDOG |
| 432 | PPARG |
| 433 | S1PR3 |
| 434 | TYMP |
| 435 | SELL |
| 436 | CXCL12 |
| 437 | CYP3A4 |
| 438 | DAP |
| 439 | DDIT3 |
| 440 | SCN5A |
| 441 | ATXN1 |
| 442 | DES |
| 443 | DMD |
| 444 | DNAH8 |
| 445 | RCAN1 |
| 446 | RELB |
| 447 | EPHX2 |
| 448 | ABCA12 |
| 449 | ACTA1 |
| 450 | ACTN2 |
| 451 | ACVR1 |
| 452 | ADAM17 |
| 453 | ADH1 |
| 454 | ADORA3 |
| 455 | AGRN |
| 456 | AGTR1A |
| 457 | AGXT |
| 458 | ALMS1 |
| 459 | ANGPTL3 |
| 460 | ANTXR1 |
| 461 | AOMS1 |
| 462 | APOA2 |
| 463 | ARL6IP6 |
| 464 | ATFB5 |
| 465 | BARD1 |
| 466 | BCL2L11 |
| 467 | CAMP |
| 468 | CASP3 |
| 469 | CD96 |
| 470 | CDC73 |
| 471 | CEP19 |
| 472 | CFH |
| 473 | CFHR1 |
| 474 | CFHR3 |
| 475 | CHDS2 |
| 476 | CLIC4 |
| 477 | CMYA1 |
| 478 | CNBP |
| 479 | COL3A1 |
| 480 | COL4A3 |
| 481 | CPT2 |
| 482 | CRIM1 |
| 483 | CTLA4 |
| 484 | CTNNB1 |
| 485 | CX3CR1 |
| 486 | CXCR4 |
| 487 | CYP27A1 |
| 488 | DCAF17 |
| 489 | DCTN1 |
| 490 | DWORF |
| 491 | EGLN1 |
| 492 | EPHB3 |
| 493 | ETFDH |
| 494 | EVC |
| 495 | F13B |
| 496 | FCGR2B |
| 497 | FCGR3 |
| 498 | FGA |
| 499 | FGF10 |
| 500 | FN1 |
| 501 | FRZB |
| 502 | FSTL1 |
| 503 | GJA5 |
| 504 | GNAI2 |
| 505 | GPR17 |
| 506 | GPX1 |
| 507 | GUCY1A1 |
| 508 | HAND2 |
| 509 | HEG1 |
| 510 | HJV |
| 511 | HOPX |
| 512 | HSD11B1 |
| 513 | HSD17B7 |
| 514 | HTRA2 |
| 515 | HTT |
| 516 | ID2 |
| 517 | IDH1 |
| 518 | IDUA |
| 519 | IL20 |
| 520 | IRX4 |
| 521 | ISL1 |
| 522 | ITGA2 |
| 523 | KCNJ3 |
| 524 | KCNK2 |
| 525 | KHK |
| 526 | KLF15 |
| 527 | KMO |
| 528 | LGV1 |
| 529 | LMNA |
| 530 | LRP8 |
| 531 | MAPK10 |
| 532 | MAPKAPK2 |
| 533 | MCM6 |
| 534 | MIR34A |
| 535 | MPVQTL2 |
| 536 | MPZ |
| 537 | MTERF4 |
| 538 | MTOR |
| 539 | MYL3 |
| 540 | MYMY1 |
| 541 | NAB1 |
| 542 | NDNF |
| 543 | NEXN |
| 544 | NFKB1 |
| 545 | NGLY1 |
| 546 | NOTCH2 |
| 547 | OR13G1 |
| 548 | PACC1 |
| 549 | PALLD |
| 550 | PCCB |
| 551 | PDE4D |
| 552 | PDGFC |
| 553 | PDGFRA |
| 554 | PDPN |
| 555 | PITX2 |
| 556 | PLEKHM2 |
| 557 | PPA2 |
| 558 | PPARGC1A |
| 559 | PRDM16 |
| 560 | PRKRA |
| 561 | PROC |
| 562 | PROS1 |
| 563 | PTPN22 |
| 564 | QTV |
| 565 | RAB3GAP2 |
| 566 | REN1 |
| 567 | RNU4ATAC |
| 568 | S100A1 |
| 569 | SCN9A |
| 570 | SERPINC1 |
| 571 | SF3B1 |
| 572 | SFRP2 |
| 573 | SLC16A1 |
| 574 | SLC19A2 |
| 575 | SLC1A3 |
| 576 | SLC25A20 |
| 577 | SLC2A1 |
| 578 | SLC2C |
| 579 | SMARCAL1 |
| 580 | SORT1 |
| 581 | SPRY1 |
| 582 | STRK1 |
| 583 | SUCNR1 |
| 584 | TET2 |
| 585 | TLL1 |
| 586 | TLR9 |
| 587 | TMEM43 |
| 588 | TNFSF4 |
| 589 | TNNC1 |
| 590 | TNNI3K |
| 591 | TREX1 |
| 592 | TRIM2 |
| 593 | UGDH |
| 594 | VHL |
| 595 | VPS54 |
| 596 | WNT5A |
| 597 | XDH |
| 598 | YRDC |
| 599 | YY1AP1 |
| 600 | ZFP687 |
