# Supplementary material for: Uncovering the Protective Mechanism of the Volatile Oil of Acorus tatarinowii against Acute Myocardial Ischemia Injury Using Network Pharmacology and Experimental Validation
Source: Evid Based Complement Alternat Med. 2021 Jun 22;2021:6630795. doi: 10.1155/2021/6630795 (PMC8241509; doi:10.1155/2021/6630795)
Supplement: Supplementary Materials — Detailed search strategy. [file 6630795.f1.zip › 6630795.f1/Supplementary Table S3.docx]

Table S3 The detail information of overlapping targets

| Number | Overlapping targets |
| --- | --- |
| 1 | NOS2 |
| 2 | PPARG |
| 3 | PPARA |
| 4 | HIF1A |
| 5 | ESR1 |
| 6 | CYP2C19 |
| 7 | HMOX1 |
| 8 | ABCC9 |
| 9 | CYP2B6 |
| 10 | HSPB1 |
| 11 | MAPK3 |
| 12 | CTSC |
| 13 | PDE4D |
| 14 | KDR |
| 15 | CASP3 |
| 16 | HRH2 |
| 17 | CYP2C9 |
| 18 | TNNT2 |
| 19 | TNF |
| 20 | SENP1 |
| 21 | P2RX7 |
| 22 | MPO |
| 23 | KCNK2 |
| 24 | APLNR |
| 25 | ADRB2 |
| 26 | UGT1A1 |
| 27 | SHH |
| 28 | TLR9 |
| 29 | NPC1L1 |
| 30 | NOS3 |
| 31 | PIK3CB |
| 32 | PIK3CA |
| 33 | MIF |
| 34 | JAK2 |
| 35 | NFKBIA |
| 36 | KCNH2 |
| 37 | ADH1C |
| 38 | ADORA2B |
| 39 | S1PR3 |
| 40 | PDE4B |
| 41 | MAPK10 |
| 42 | CETP |
| 43 | PRKCE |
| 44 | PER2 |
| 45 | MAPK14 |
| 46 | IL6ST |
| 47 | CYP3A4 |
| 48 | PPAR1 |
| 49 | EPHX2 |
| 50 | CCR5 |
| 51 | ADORA3 |
| 52 | ADORA1 |
| 53 | STAT3 |
| 54 | SCN5A |
| 55 | PTGS2 |
