# Supplementary material for: Uncovering the Protective Mechanism of the Volatile Oil of Acorus tatarinowii against Acute Myocardial Ischemia Injury Using Network Pharmacology and Experimental Validation
Source: Evid Based Complement Alternat Med. 2021 Jun 22;2021:6630795. doi: 10.1155/2021/6630795 (PMC8241509; doi:10.1155/2021/6630795)
Supplement: Supplementary Materials — Detailed search strategy. [file 6630795.f1.zip › 6630795.f1/Supplementary Table S4.docx]

Table S4 The topological parameters of overlapping targets

| Number | Target name | Degree | Betweenness | Proximity centrality | Average shortest path length |
| --- | --- | --- | --- | --- | --- |
| 1 | ABCC9 | 1 | 0.00000000 | 0.26443769 | 3.78160920 |
| 2 | ADH1C | 3 | 0.00275170 | 0.29292929 | 3.41379310 |
| 3 | ADORA1 | 1 | 0.00000000 | 0.21695761 | 4.60919540 |
| 4 | ADORA2B | 2 | 0.00002700 | 0.28524590 | 3.50574713 |
| 5 | ADORA3 | 5 | 0.03396399 | 0.32830189 | 3.04597701 |
| 6 | ADRB2 | 1 | 0.00000000 | 0.27619048 | 3.62068966 |
| 7 | APLNR | 1 | 0.00000000 | 0.27619048 | 3.62068966 |
| 8 | CASP3 | 2 | 0.00002700 | 0.28524590 | 3.50574713 |
| 9 | CCR5 | 5 | 0.02517872 | 0.29896907 | 3.34482759 |
| 10 | CETP | 3 | 0.00121363 | 0.30526316 | 3.27586207 |
| 11 | CTSC | 1 | 0.00000000 | 0.23200000 | 4.31034483 |
| 12 | CYP2B6 | 1 | 0.00000000 | 0.27272727 | 3.66666667 |
| 13 | CYP2C19 | 7 | 0.03276698 | 0.33852140 | 2.95402299 |
| 14 | CYP2C9 | 5 | 0.01021447 | 0.33079848 | 3.02298851 |
| 15 | CYP3A4 | 2 | 0.00198292 | 0.30960854 | 3.22988506 |
| 16 | EPHX2 | 8 | 0.02784248 | 0.33852140 | 2.95402299 |
| 17 | ESR1 | 16 | 0.30377566 | 0.47027027 | 2.12643678 |
| 18 | HIF1A | 7 | 0.06240619 | 0.31868132 | 3.13793103 |
| 19 | HMOX1 | 1 | 0.00000000 | 0.26443769 | 3.78160920 |
| 20 | HRH2 | 1 | 0.00000000 | 0.26283988 | 3.80459770 |
| 21 | HSPB1 | 1 | 0.00000000 | 0.21588089 | 4.63218391 |
| 22 | IL6ST | 1 | 0.00000000 | 0.27974277 | 3.57471264 |
| 23 | JAK2 | 5 | 0.01128432 | 0.32830189 | 3.04597701 |
| 24 | KCNH2 | 5 | 0.01934572 | 0.33590734 | 2.97701149 |
| 25 | KCNK2 | 1 | 0.00000000 | 0.27619048 | 3.62068966 |
| 26 | KDR | 2 | 0.00002700 | 0.28524590 | 3.50574713 |
| 27 | MAPK10 | 3 | 0.00121363 | 0.30526316 | 3.27586207 |
| 28 | MAPK14 | 5 | 0.01010923 | 0.33079848 | 3.02298851 |
| 29 | MAPK3 | 3 | 0.00081056 | 0.29096990 | 3.43678161 |
| 30 | MIF | 3 | 0.00104945 | 0.29491525 | 3.39080460 |
| 31 | MPO | 8 | 0.04989834 | 0.37339056 | 2.67816092 |
| 32 | NFKBIA | 2 | 0.00159935 | 0.28338762 | 3.52873563 |
| 33 | NOS2 | 8 | 0.06101713 | 0.37021277 | 2.70114943 |
| 34 | NOS3 | 4 | 0.01277536 | 0.33333333 | 3.00000000 |
| 35 | NPC1L1 | 5 | 0.01687874 | 0.30742049 | 3.25287356 |
| 36 | P2RX7 | 10 | 0.07025812 | 0.39013453 | 2.56321839 |
| 37 | PARP1 | 6 | 0.01389097 | 0.33079848 | 3.02298851 |
| 38 | PDE4B | 4 | 0.00373541 | 0.31182796 | 3.20689655 |
| 39 | PDE4D | 2 | 0.00002700 | 0.28524590 | 3.50574713 |
| 40 | PER2 | 1 | 0.00000000 | 0.27974277 | 3.57471264 |
| 41 | PIK3CA | 1 | 0.00000000 | 0.27272727 | 3.66666667 |
| 42 | PIK3CB | 1 | 0.00000000 | 0.27272727 | 3.66666667 |
| 43 | PPARα | 12 | 0.12877342 | 0.31182796 | 3.20689655 |
| 44 | PPARG | 8 | 0.09192557 | 0.34387352 | 2.90804598 |
| 45 | PRKCE | 1 | 0.00000000 | 0.27974277 | 3.57471264 |
| 46 | PTGS2 | 9 | 0.04705197 | 0.36099585 | 2.77011494 |
| 47 | S1PR3 | 1 | 0.00000000 | 0.26283988 | 3.80459770 |
| 48 | SCN5A | 3 | 0.00104945 | 0.29491525 | 3.39080460 |
| 49 | SENP1 | 1 | 0.00000000 | 0.27619048 | 3.62068966 |
| 50 | SHH | 2 | 0.02298851 | 0.25217391 | 3.96551724 |
| 51 | STAT3 | 3 | 0.00217929 | 0.30313589 | 3.29885057 |
| 52 | TLR9 | 2 | 0.00159935 | 0.28338762 | 3.52873563 |
| 53 | TNF | 1 | 0.00000000 | 0.27619048 | 3.62068966 |
| 54 | TNNT2 | 1 | 0.00000000 | 0.27619048 | 3.62068966 |
| 55 | UGT1A1 | 1 | 0.00000000 | 0.25072046 | 3.98850575 |
