# Supplementary material for: Uncovering the Protective Mechanism of the Volatile Oil of Acorus tatarinowii against Acute Myocardial Ischemia Injury Using Network Pharmacology and Experimental Validation
Source: Evid Based Complement Alternat Med. 2021 Jun 22;2021:6630795. doi: 10.1155/2021/6630795 (PMC8241509; doi:10.1155/2021/6630795)
Supplement: Supplementary Materials — Detailed search strategy. [file 6630795.f1.zip › 6630795.f1/Supplementary Table S5.docx]

Table S5 The topological parameters of chemistry components of VOA

| Number | chemical composition | Degree | Betweenness | Proximity centrality | Average shortest path length |
| --- | --- | --- | --- | --- | --- |
| VOA1 | α-Pinene | 5 | 0.04649524 | 0.37991266 | 2.63218391 |
| VOA2 | Camphene | 1 | 0.00000000 | 0.24233983 | 4.12643678 |
| VOA3 | β-Pinene | 1 | 0.00000000 | 0.24233983 | 4.12643678 |
| VOA4 | o-Cymene | 1 | 0.00000000 | 0.23835616 | 4.19540230 |
| VOA5 | D-Limonene | 5 | 0.04465390 | 0.34939759 | 2.86206897 |
| VOA6 | γ-Terpinene | 3 | 0.01034377 | 0.29692833 | 3.36781609 |
| VOA7 | Camphor | 6 | 0.02372450 | 0.31407942 | 3.18390805 |
| VOA8 | endo-Borneol | 4 | 0.06961954 | 0.33333333 | 3.00000000 |
| VOA9 | Estragole | 12 | 0.18329422 | 0.37991266 | 2.63218391 |
| VOA10 | Longicyclene | 1 | 0.00000000 | 0.23835616 | 4.19540230 |
| VOA11 | Caryophyllene | 1 | 0.00000000 | 0.23835616 | 4.19540230 |
| VOA12 | γ-Muurolene | 3 | 0.01994114 | 0.36099585 | 2.77011494 |
| VOA13 | 1H-Cyclopropa[a]naphthalene, 1a,2,3,5,6,7,7a,7b-octahydro-1,1,7,7a-tetramethyl-, [1aR-(1a.alpha.,7.alpha.,7a.alpha.,7b.alpha.)]- | 3 | 0.01994114 | 0.36099585 | 2.77011494 |
| VOA14 | Methyl isoeugenol | 13 | 0.08443063 | 0.37339056 | 2.67816092 |
| VOA15 | 1,3,5-Cycloheptatriene, 2,4-diethyl-7,7-dimethyl- | 3 | 0.02760776 | 0.27619048 | 3.62068966 |
| VOA16 | Cyclohexyl-2，4-dimethylbenzene ketone | 7 | 0.02113915 | 0.35802469 | 2.79310345 |
| VOA17 | α-Gurjunene | 1 | 0.00000000 | 0.23835616 | 4.19540230 |
| VOA18 | Shyobunone | 3 | 0.00096111 | 0.30103806 | 3.32183908 |
| VOA19 | 1,6,10-Dodecatrien-3-ol, 3,7,11-trimethyl- | 13 | 0.12632371 | 0.38666667 | 2.58620690 |
| VOA20 | Benzene, 1,2,3-trimethoxy-5-(2-propenyl)- | 13 | 0.06413478 | 0.37991266 | 2.63218391 |
| VOA21 | Hexadecane | 1 | 0.00000000 | 0.25663717 | 3.89655172 |
| VOA22 | Tumerone | 13 | 0.07315755 | 0.35510204 | 2.81609195 |
| VOA23 | β-asarone | 18 | 0.09676394 | 0.39013453 | 2.56321839 |
| VOA24 | Cyclohexanone, 6-furfurylidene-2,2,3-trimethyl- | 3 | 0.02345010 | 0.30103806 | 3.32183908 |
| VOA25 | Naphthalene, 1,2,3,5,6,7,8,8a-octahydro-1,8a-dimethyl-7-(1-methylethenyl)-, [1R-(1.alpha.,7.beta.,8a.alpha.)]- | 4 | 0.03054834 | 0.37021277 | 2.70114943 |
| VOA26 | Spiro[4.5]dec-6-en-8-one, 1,7-dimethyl-4-(1-methylethyl)- | 10 | 0.05057624 | 0.37662338 | 2.65517241 |
| VOA27 | α-asarone | 19 | 0.11081604 | 0.39366516 | 2.54022989 |
| VOA28 | 2H-Cyclopropa[a]naphthalen-2-one, 1,1a,4,5,6,7,7a,7b-octahydro-1,1,7,7a-tetramethyl-, (1a.alpha.,7.alpha.,7a.alpha.,7b.alpha.)- | 9 | 0.04375949 | 0.37339056 | 2.67816092 |
| VOA29 | Cyclolongifolene oxide, dehydro- | 1 | 0.00000000 | 0.20185615 | 4.95402299 |
| VOA30 | 2,4,6-Octatriene, 2,6-dimethyl- | 3 | 0.02714393 | 0.27444795 | 3.64367816 |
| VOA31 | Bicyclo[3.1.1]hept-3-en-2-one, 4,6,6-trimethyl-, (1S)- | 9 | 0.07141655 | 0.37339056 | 2.67816092 |
| VOA32 | Isocalamendiol | 9 | 0.05720979 | 0.35802469 | 2.79310345 |
| VOA33 | 5(1H)-Azulenone, 2,4,6,7,8,8a-hexahydro-3,8-dimethyl-4-(1-methylethylidene)-, (8S-cis)- | 1 | 0.00000000 | 0.23076923 | 4.33333333 |
