# Supplementary material for: Uncovering the Protective Mechanism of the Volatile Oil of Acorus tatarinowii against Acute Myocardial Ischemia Injury Using Network Pharmacology and Experimental Validation
Source: Evid Based Complement Alternat Med. 2021 Jun 22;2021:6630795. doi: 10.1155/2021/6630795 (PMC8241509; doi:10.1155/2021/6630795)
Supplement: Supplementary Materials — Detailed search strategy. [file 6630795.f1.zip › 6630795.f1/Supplementary Table S6.docx]

Table S6 Enriched KEGG pathways of overlapping targets

| Description | P value | P adjust | Q value | Count |
| --- | --- | --- | --- | --- |
| AGE-RAGE signaling pathway in diabetic complications | 3.95E-11 | 8.13E-09 | 3.24E-09 | 11 |
| Kaposi sarcoma-associated herpesvirus infection | 1.76E-10 | 1.82E-08 | 7.23E-09 | 13 |
| Sphingolipid signaling pathway | 2.68E-10 | 1.84E-08 | 7.32E-09 | 11 |
| Toxoplasmosis | 2.93E-09 | 1.51E-07 | 6.01E-08 | 10 |
| VEGF signaling pathway | 4.20E-09 | 1.73E-07 | 6.90E-08 | 8 |
| PD-L1 expression and PD-1 checkpoint pathway in cancer | 5.97E-09 | 2.05E-07 | 8.17E-08 | 9 |
| Prolactin signaling pathway | 1.69E-08 | 4.98E-07 | 1.98E-07 | 8 |
| Chagas disease (American trypanosomiasis) | 2.20E-08 | 5.65E-07 | 2.25E-07 | 9 |
| Insulin resistance | 3.34E-08 | 7.64E-07 | 3.04E-07 | 9 |
| TNF signaling pathway | 3.92E-08 | 8.08E-07 | 3.22E-07 | 9 |
| Proteoglycans in cancer | 6.92E-08 | 1.30E-06 | 5.17E-07 | 11 |
| Hepatitis B | 1.01E-07 | 1.73E-06 | 6.90E-07 | 10 |
| Apoptosis | 2.48E-07 | 3.92E-06 | 1.56E-06 | 9 |
| HIF-1 signaling pathway | 2.86E-07 | 4.21E-06 | 1.68E-06 | 8 |
| Toll-like receptor signaling pathway | 3.88E-07 | 4.99E-06 | 1.99E-06 | 8 |
| C-type lectin receptor signaling pathway | 3.88E-07 | 4.99E-06 | 1.99E-06 | 8 |
| Th17 cell differentiation | 4.83E-07 | 5.81E-06 | 2.31E-06 | 8 |
| Type II diabetes mellitus | 5.35E-07 | 5.81E-06 | 2.31E-06 | 6 |
| Leishmaniasis | 5.35E-07 | 5.81E-06 | 2.31E-06 | 7 |
| cAMP signaling pathway | 1.27E-06 | 1.31E-05 | 5.21E-06 | 10 |
